# Emergent superconductivity in topological-kagome-magnet/metal heterostructures

**DOI:** 10.1038/s41467-023-42779-1

**Published:** 2023-11-02

**Authors:** He Wang, Yanzhao Liu, Ming Gong, Hua Jiang, Xiaoyue Gao, Wenlong Ma, Jiawei Luo, Haoran Ji, Jun Ge, Shuang Jia, Peng Gao, Ziqiang Wang, X. C. Xie, Jian Wang

**Affiliations:** 1 International Center for Quantum Materials, School of Physics, Peking University, Beijing, 100871 China; 2 Center for Quantum Physics and Intelligent Sciences, Department of Physics, Capital Normal University, Beijing, 100048 China; 3 Institute for Advanced Study, Soochow University, Suzhou, 215006 China; 4 Department of Physics, Boston College, Chestnut Hill, MA 02467 USA; 5 Hefei National Laboratory, Hefei, 230088 China; 6 Institute for Nanoelectronic Devices and Quantum Computing, Fudan University, Shanghai, 200433 China; 7 Collaborative Innovation Center of Quantum Matter, Beijing, 100871 China

**Keywords:** Superconducting properties and materials, Surfaces, interfaces and thin films, Topological matter, Superconducting properties and materials, Topological matter

## Abstract

Itinerant kagome lattice magnets exhibit many novel correlated and topological quantum electronic states with broken time-reversal symmetry. Superconductivity, however, has not been observed in this class of materials, presenting a roadblock in a promising path toward topological superconductivity. Here, we report that novel superconductivity can emerge at the interface of kagome Chern magnet TbMn_6_Sn_6_ and metal heterostructures when elemental metallic thin films are deposited on either the top (001) surface or the side surfaces. Superconductivity is also successfully induced and systematically studied by using various types of metallic tips on different TbMn_6_Sn_6_ surfaces in point-contact measurements. The anisotropy of the superconducting upper critical field suggests that the emergent superconductivity is quasi-two-dimensional. Remarkably, the interface superconductor couples to the magnetic order of the kagome metal and exhibits a hysteretic magnetoresistance in the superconducting states. Taking into account the spin-orbit coupling, the observed interface superconductivity can be a surprising and more realistic realization of the *p*-wave topological superconductors theoretically proposed for two-dimensional semiconductors proximity-coupled to *s*-wave superconductors and insulating ferromagnets. Our findings of robust superconductivity in topological-Chern-magnet/metal heterostructures offer a new direction for investigating spin-triplet pairing and topological superconductivity.

## Abstract (web summary)

The authors deposit non-superconducting metallic thin films on surfaces of the kagome Chern magnet TbMn_6_Sn_6_ and observe emergent superconductivity even though neither component is a superconductor. Furthermore, the superconducting state is quasi-two-dimensional and coexists with ferromagnetism, consistent with possible spin-triplet pairing and topological superconductivity.

## Introduction

The heterostructure interface between two different materials has become a frontier to generate and investigate emergent quantum phases^1–12^ such as the quantum Hall effect^12^ and novel superconductivity^1–11^. In the heterostructures formed by topological materials, the superconducting state may inherit topological properties and show great potential for non-Abelian defect excitations for topological fault-tolerant quantum computation^2,13–17^.

The kagome magnet TbMn_6_Sn_6_ has recently been discovered to have Chern-gapped Dirac fermions with dissipationless chiral edge states, coexisting with ferrimagnetism below 423 K^18^. Thus, the long-sought-after time-reversal symmetry (TRS) broken spin-triplet superconducting state^19–22^ and topological superconductivity^14,15,23–27^ would potentially be realized if TbMn_6_Sn_6_ exhibited a superconducting ground state. However, superconductivity has so far not been observed in TbMn_6_Sn_6_ and other itinerant kagome magnets.

In this work, we report the emergent superconductivity at the interface between TbMn_6_Sn_6_ and non-superconducting metals. After depositing metallic films (paramagnetic metal: Au and Ag, ferromagnetic metal: Ni) on the surfaces of TbMn_6_Sn_6_ single crystals, superconductivity is observed by standard transport measurements. The hysteretic behavior in the superconducting state suggests that the superconductivity is coupled with the magnetization of TbMn_6_Sn_6_. Furthermore, by pressing metallic tips (paramagnetic tips: PtIr, Au, Ag; ferromagnetic tip: Ni) onto the crystal surfaces of the samples, a superconducting phase inheriting the magnetic properties of TbMn_6_Sn_6_ is also induced and characterized by point-contact spectra (PCS). The structural and elemental mappings show the TbMn_6_Sn_6_ sample near the interface has a naturally formed degraded layer possessing polycrystalline TbMn_6_Sn_6_. Since TbMn_6_Sn_6_ is a magnetic Chern metal^18^, the broken TRS and emergent superconducting states at the interface make the TbMn_6_Sn_6_/metal heterostructure an ideal system to realize topological superconductivity^26,27^, which we demonstrate using a simple theoretical model.

## Results

### The transport and magnetic properties of TbMn_6_Sn_6_ samples

**Fig. 1:**
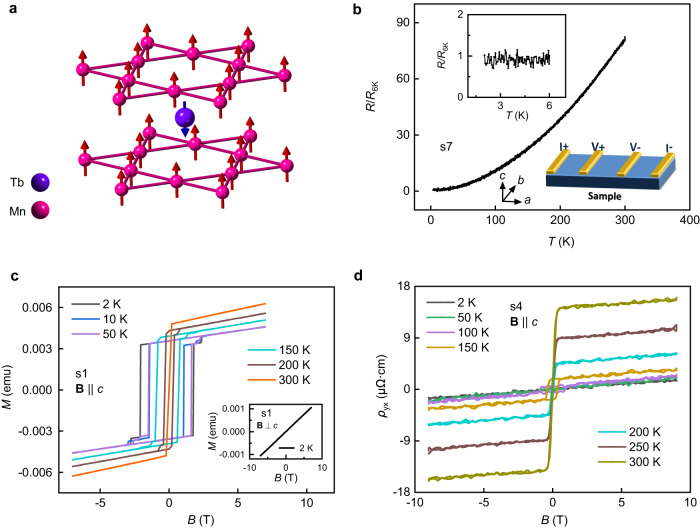
Transport and magnetic properties of TbMn_6_Sn_6_ samples. **a** The schematic of the magnetic structure of manganese (pink) and terbium (purple) atoms in TbMn_6_Sn_6_. **b** The temperature dependence of normalized resistance *R*/*R*_6K_. Related resistance is measured by the standard four-electrode method. Upper inset: the zoom-in of *R*/*R*_6K_ - *T* curve below *T* < 6 K. Lower inset: the schematic of the standard four-electrode configuration. **c** The out-of-plane (**B** | |c) magnetization curves at different temperatures. Inset: the in-plane (**B**⊥*c*) magnetization curve taken at 2 K. **d** The magnetic field (**B** | |*c*) evolution of Hall resistivity at different temperatures.

The high-quality TbMn_6_Sn_6_ single crystals are grown via a flux method^18,28^. The TbMn_6_Sn_6_ single crystals crystallize in a hexagonal structure with space group P6/mmm (see Supplementary Fig. 1) and show ferrimagnetic behavior below an ordering temperature ≈ 423 K^18,28^. The atomic and magnetic structures are shown in Fig. 1a. In a single unit cell, Mn atoms with out-of-plane magnetic order form two kagome layers, and one Tb atom with opposite magnetic moment locates between the two Mn layers. The temperature dependence of the normalized longitudinal resistance (*R/R*_6K_) of the TbMn_6_Sn_6_ sample is shown in Fig. 1b. The *R/R*_6K_-*T* curve exhibits a typically metallic behavior, and no extra features are detected at low temperatures (inset of Fig. 1b), consistent with early work^18^. The magnetic properties of TbMn_6_Sn_6_ are investigated by the electrical transport and magnetization measurements. As shown in Fig. 1c, the magnetization as a function of the magnetic field exhibits explicit hysteresis loops with a sizeable coercive field of about 1.8 T (at 2 K) when the magnetic field is applied along the *c* axis (perpendicular to the kagome layer). However, under the in-plane magnetic field, the magnetization shows linear magnetic field dependence (inset of Fig. 1c). The magnetization results show that the TbMn_6_Sn_6_ crystal has out-of-plane magnetic order, which is consistent with the previous report^18^. The anomalous Hall effect has also been detected by transport measurements (Fig. 1d).

### The emergent superconductivity in TbMn_6_Sn_6_/metallic film heterostructures revealed by transport measurements

**Fig. 2:**
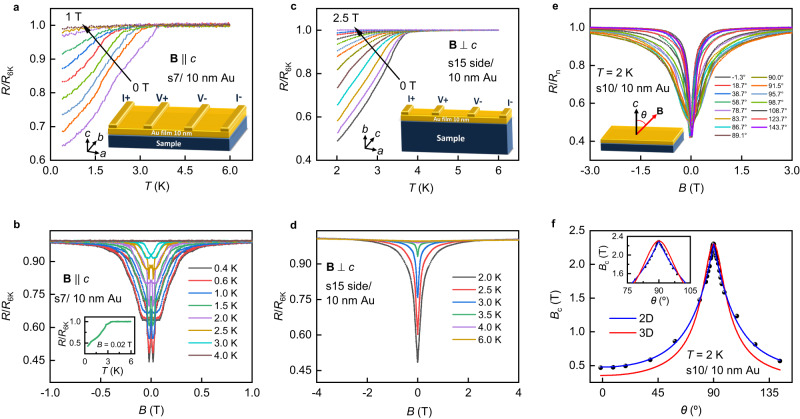
Emergent superconductivity at the interface between the TbMn_6_Sn_6_ sample and the Au film. **a** The evidence of superconductivity for Au film-coated (001) surface of TbMn_6_Sn_6_ single-crystal (s7) detected by the standard four-electrode method. The curves are measured at **B** = 0 T, 0.12 T, 0.145 T, 0.175 T, 0.21 T, 0.25 T, 0.3 T, 0.45 T, 0.7 T, and 1 T, respectively. The magnetic field is applied along the out-of-plane direction (**B** | |*c* axis). Inset: the schematic of the standard four-electrode measurements for the TbMn_6_Sn_6_/Au film sample. **b** The magnetoresistance measurements of s7 capped with 10 nm Au film at different temperatures (**B** | |*c* axis). Inset: the temperature dependence of normalized resistance at 0.02 T with a drop down to nearly 40% of *R*_6K_. **c** The evidence of superconductivity for Au film-coated side surface of a TbMn_6_Sn_6_ single-crystal (s15) detected by the standard four-electrode method. The curves are measured at 0 T, 0.02 T, 0.05 T, 0.09 T, 0.14 T, 0.23 T, 0.37 T, 0.48 T, 0.65 T, 0.85 T, 1.2 T, 1.5 T, and 2.5 T, respectively. The magnetic field is applied perpendicular to the side surface (**B**⊥*c* axis). Inset: the schematic of the standard four-electrode measurements for the TbMn_6_Sn_6_/Au film sample. **d** The magnetoresistance measurements of s15 capped with 10 nm Au film on the side surface at different temperatures (**B**⊥*c* axis). **e** The angular dependent magnetoresistance curves of a TbMn_6_Sn_6_ sample (s10) capped with 10 nm Au film at 2 K. Here *θ* is the angle between the magnetic field and *c*-axis of the TbMn_6_Sn_6_ sample. *R*_n_ is the magnetoresistance at 3 T when **B** | |*c* axis. **f** Angular dependence of the critical magnetic field *B*_c_(*θ*) of s10 capped with 10 nm Au film. Inset shows the zoom-in of the *B*_c_(*θ*) around 90°. The blue curve is the fitting with two-dimensional (2D) Tinkham model (*B*_c_ (*θ*) sin (*θ*)/*B*_c//_)^2^ + |*B*_c_(*θ*)cos(θ)/*B*_c⊥_| = 1 and the red curve is the fitting with the three-dimensional (3D) anisotropic mass model *B*_c_(*θ*) = *B*_c//_/(sin^2^(*θ*)+*γ*^2^cos^2^(*θ*))^1/2^ with *γ* = *B*_c//_/*B*_c⊥_. *B*_c_ is defined as the magnetic field corresponding to 95% normal resistance.

Surprisingly, when the (001) surface of the sample is capped with a 10 nm Au film, a significant resistance drop down to nearly 60% of *R*_6K_ is observed at around 3.6 K (Fig. 2a). The drop in *R/R*_6K_-*T* curves is reminiscent of the superconducting transition. When the magnetic field is applied along the *c* axis, this drop weakens and finally disappears, confirming the emergence of superconductivity at the interface between the Au film and (001) surface of TbMn_6_Sn_6_. Even the magnetic Ni film deposited on the (001) surface of the sample shows superconducting signals with zero-field onset *T*_c_ ≈ 3.5 K (see Supplementary Fig. 2), close to that of heterostructures capped with the Au film. The observation of a similar superconducting state for both the magnetic Ni and the nonmagnetic Au capping films on the ferrimagnet TbMn_6_Sn_6_ substrate suggests that TRS is broken in the interface superconductivity.

The nature of the interface superconductivity is further studied by transport measurements when the side surface of the TbMn_6_Sn_6_ crystal is capped with a 10 nm Au film. Figure 2c shows one set of typical results obtained from a sample labeled s15. At zero magnetic field, the resistance drops down to less than 50% of *R*_6K_, which can be suppressed by external magnetic fields, showing the signature of superconductivity. To probe the interplay between magnetism and superconductivity, we characterize the magnetic anisotropy of TbMn_6_Sn_6_/Au. For the heterostructure formed by depositing the Au film on the (001) top surface of TbMn_6_Sn_6_, the magnetoresistance (MR) curves show hysteretic behavior when the magnetic field is perpendicular to the Au film (Fig. 2b, **B** | |*c* axis). However, the hysteresis in MR curves disappears when the Au film is capped on the side surface of TbMn_6_Sn_6_ (Fig. 2d) and the magnetic field is applied perpendicularly to the side surface (**B**⊥*c* axis). Remarkably, the hysteresis anisotropy observed in the interface superconducting phase of TbMn_6_Sn_6_/Au film heterostructure qualitatively follows that of the anisotropic magnetization in the non-superconducting bulk ferrimagnet TbMn_6_Sn_6_ (Fig. 1c). In addition, the resistance drop becomes more notable after the magnetization treatment on the TbMn_6_Sn_6_/Au heterostructure (Supplementary Fig. 3), indicating that magnetism is favorable for the formation of superconductivity. The ferromagnetic exchange coupling is known to frustrate the time-reversal invariant spin-singlet Cooper pairs and promote Cooper pairs having spin-triplet pairing symmetry^29^. It is thus natural to expect that the emergent interface superconductivity in the TbMn_6_Sn_6_/Au film heterostructure has at least a significant triplet-pairing (such as *p*-wave) component.

Furthermore, the anisotropy of the emergent superconductivity is revealed by angular-dependent transport measurements. As shown in Fig. 2e–f, a cusp-like peak is clearly observed in the angular dependence of the critical magnetic field *B*_c_(*θ*) of TbMn_6_Sn_6_/Au heterostructure (s10), here *θ* is the angle between the magnetic field and *c*-axis of TbMn_6_Sn_6_ sample. The peak feature can be well fitted by the two-dimensional (2D) Tinkham model and deviates from the three-dimensional (3D) anisotropic mass model^30^, demonstrating quasi-2D superconductivity at the TbMn_6_Sn_6_/Au heterostructure. Moreover, the temperature dependence of the critical field, presented in Supplementary Figs. 4a and 4b, show the (*T*_c_-*T*)^1/2^-dependence of *B*_c//_(*T*) (magnetic field applied perpendicular to *c*-axis) and *T*-linear dependence of *B*_c⊥_(*T*) (magnetic field applied along the *c*-axis) near *T*_c_, respectively, which are also consistent with the phenomenological 2D Ginzburg-Landau (GL) model^31^. The quasi-2D superconductivity of the TbMn_6_Sn_6_/Au heterostructure further supports that the observed superconductivity is emergent at the interface between TbMn_6_Sn_6_ and deposited metal film.

To probe the robustness of the observed interface superconductivity, the (001) top surface of samples is coated with silver epoxy, and transport properties are measured in an applied magnetic field. As shown in Supplementary Fig. 5, the results of TbMn_6_Sn_6_/silver epoxy confirm the robustness of the emergent interface superconductivity between two non-superconducting partners, the topological Chern magnet and metallic films. A control experiment is further carried out to test the influence of the interface condition on the emergent superconductivity. The silver film is evaporated on TbMn_6_Sn_6_ by sputtering to form a TbMn_6_Sn_6_/Ag film heterostructure with a better interface than the TbMn_6_Sn_6_/silver epoxy heterostructure. As shown in Supplementary Fig. 6, the observed superconducting signal in TbMn_6_Sn_6_/Ag film heterostructure is more notable than that induced by the silver epoxy, indicating that good contact between the metallic film and TbMn_6_Sn_6_ is conducive to the formation of superconductivity. Due to the non-van der Waals nature of TbMn_6_Sn_6_ crystals, it is challenging to obtain a large area of atomic-level flat surfaces from the micrometer-scale TbMn_6_Sn_6_ single crystals. As a result, when depositing a metallic film onto the TbMn_6_Sn_6_ surface, it is difficult to achieve a uniform and consistent contact condition, which may lead to inhomogeneous superconductivity with non-zero residual resistance at low temperatures.

### The emergent superconductivity in TbMn_6_Sn_6_/metal heterostructures confirmed by point-contact measurements

**Fig. 3:**
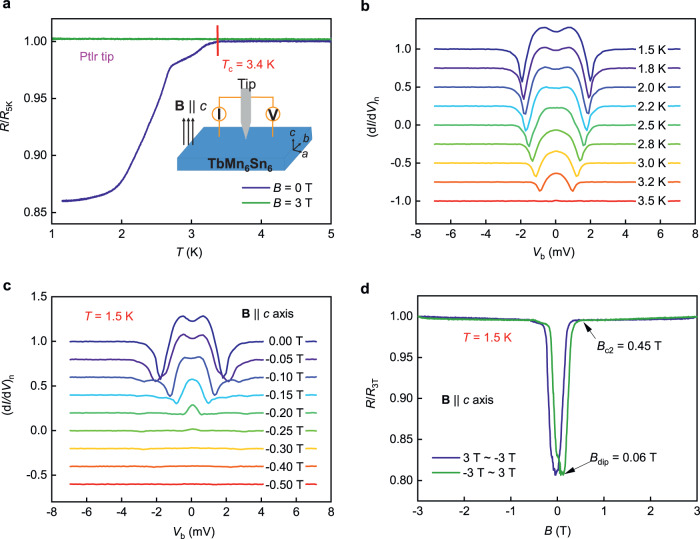
The evidence of superconductivity at the point contact (PC) by pressing the PtIr tip onto the (001) surface of TbMn_6_Sn_6_. **a** The temperature dependence of the normalized PC resistance (*R*/*R*_5K_) at zero bias with (green curve, *B* = 3 T) or without (blue, *B* = 0 T) applying the magnetic field. Inset: the schematic of the PC configuration, the magnetic field is applied along the out-of-plane direction (**B** | |*c* axis). **b**, **c** The temperature and magnetic field dependence of the normalized point-contact spectra (PCS). The PCS curves are shifted vertically for clarity. The applied magnetic field is ramping from 0 T to −0.5 T at 1.5 K. **d** The magnetoresistance measurements of the PC at 1.5 K. The PC resistance in the normal state at 5 K is 10.7 Ω.

To further investigate the emergent superconductivity at the TbMn_6_Sn_6_/metal heterostructure, different metallic tips are used to carry out the point-contact (PC) measurements on TbMn_6_Sn_6_ samples in a cryogenic system. The main results of one typical PC state obtained by pressing a PtIr tip onto the (001) surface of the sample (s1) are shown in Fig. 3. Figure 3a shows the temperature dependence of the normalized PC resistance (*R*/*R*_5K_) with and without applying the magnetic field. The differential PC resistance (*R*) is obtained by using the standard lock-in technique in a quasi-four-electrode PC configuration (the inset of Fig. 3a). When the external magnetic field is zero, a resistance drop with a transition temperature of 3.4 K (blue curve) is observed, consistent with the standard four-electrode transport measurements for the superconducting TbMn_6_Sn_6_/Au film heterostructures (Fig. 2a). This PC resistance versus temperature curve seems to undergo a two-step transition, which may attribute to the different superconducting phases triggered by complex interfacial conditions in the contact region. The magnetic field of 3 T along the *c* axis can suppress the resistance drop (green curve in Fig. 3a), which further indicates the emergence of superconductivity at the interface between the PtIr tip and the (001) surface of TbMn_6_Sn_6_. The temperature and magnetic field evolutions of the PCS are presented in Fig. 3b and c, respectively. The PCS at 1.5 K (marked in Fig. 3b) shows clearly two conductance peaks at ±0.6 mV. For a PC, the two conductance peaks are usually taken as the hallmark of Andreev reflection processes at the interface between a normal metal and a superconductor^32^. With the increase of temperature or magnetic field, the two conductance peaks are gradually suppressed, consistent with the signature of weakening superconductivity, confirming the emergence of superconductivity at the TbMn_6_Sn_6_/PtIr tip PC interface.

Interestingly, the MR curve at 1.5 K (Fig. 3d) shows a notable hysteresis loop when applying the magnetic field along the TbMn_6_Sn_6_ *c* axis (**B** | |c axis). Furthermore, this hysteresis loop disappears when the magnetic field is applied along the in-plane direction (**B**⊥*c* axis), as shown in Supplementary Fig. 7. Thus, the MR curves show a similar magnetic anisotropy to the bulk ferrimagnetic TbMn_6_Sn_6_ (Fig. 1c) and TbMn_6_Sn_6_/metallic film heterostructure (Fig. 2), suggesting that the superconducting state in the PC configuration couples to the magnetization along the out-of-plane direction^33,34^ and thus breaks TRS, in agreement with the interface superconductivity in TbMn_6_Sn_6_/metallic film heterostructures discussed above (Fig. 2). We note in passing that the superconductivity can also be induced on the side surface of TbMn_6_Sn_6_ with PtIr tips. The results for one such PC can be found in Supplementary Fig. 8. Similar to the TbMn_6_Sn_6_/Ni film heterostructure (see Supplementary Fig. 2), the superconductivity is also successfully induced by using ferromagnetic Ni tips for PC on TbMn_6_Sn_6_. Related results and discussions can be referred to Supplementary Fig. 9, Fig. 10, and Text I.

To test the reproducibility of the experimental observations presented above, we have probed 92 PC positions and 170 PC states on the different surfaces of two TbMn_6_Sn_6_ samples from the same batch using various metallic tips, including PtIr, Au, Ni, and Ag (see Supplementary Fig. 11a). All these non-superconducting tips are found to induce superconductivity on either the top (001) or side surfaces of TbMn_6_Sn_6_. The statistics of *T*_c_ versus the tip materials (see Supplementary Fig. 11b) show no significant variations in the maximum *T*_c_ values, whether the tip materials are relatively hard (PtIr tip) or soft (Au tip), paramagnetic or ferromagnetic. These striking observations point to a universal and highly robust interface superconducting state in TbMn_6_Sn_6_/metal heterostructures.

### The structural and elemental analyses of the interface of TbMn_6_Sn_6_/metal heterostructure

**Fig. 4:**
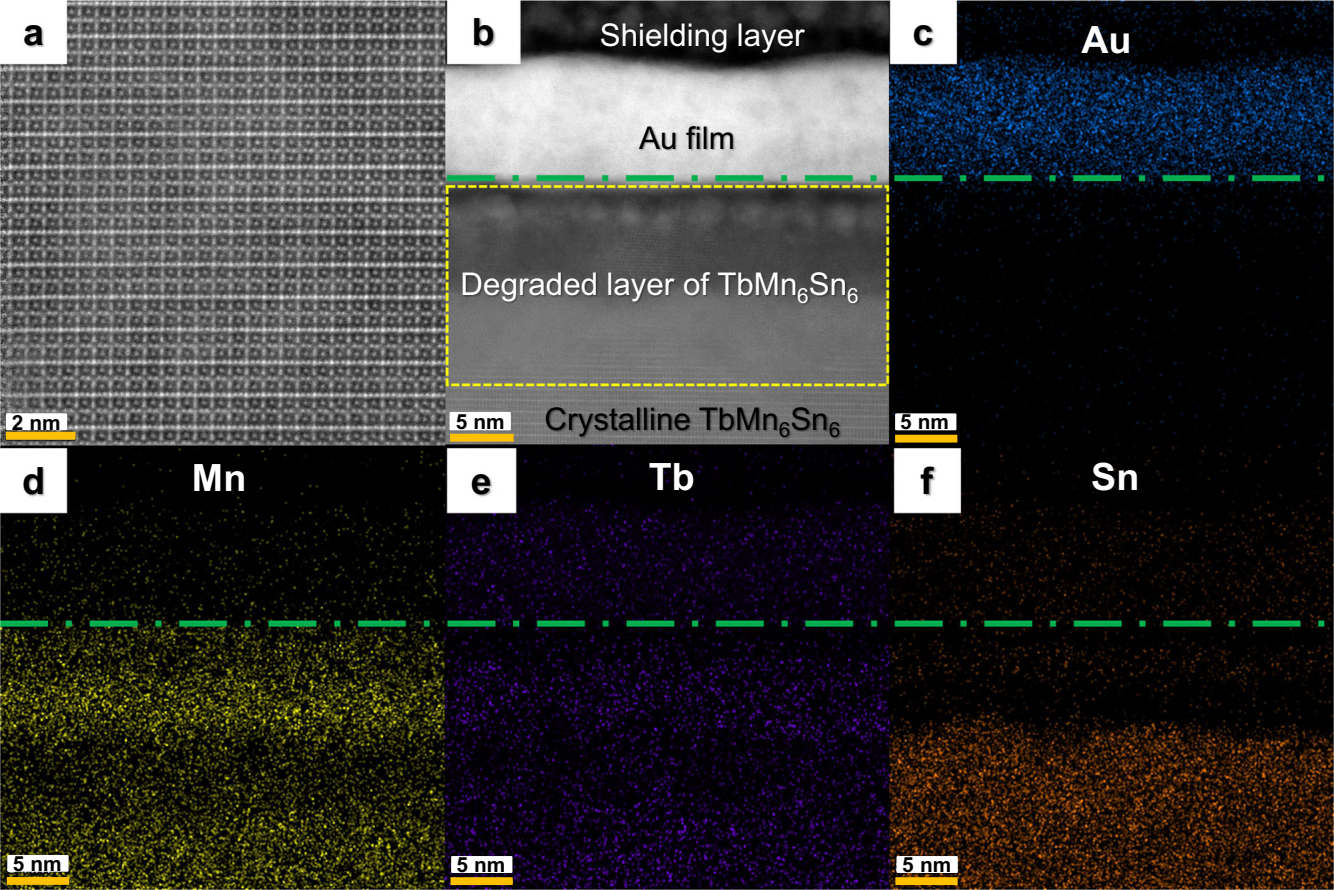
Structural images of the TbMn_6_Sn_6_ single crystal and elemental mappings of the TbMn_6_Sn_6_/Au heterostructure. **a**, **b** The cross-sectional HAADF STEM images of the TbMn_6_Sn_6_ single crystal and the TbMn_6_Sn_6_/Au heterostructure (s8). The heterostructure is fabricated by depositing 10 nm Au film on the (001) surface of the TbMn_6_Sn_6_ single crystal. **c-f** The energy-dispersive X-ray spectroscopy (EDS) mappings of Au, Mn, Tb, and Sn elements distribution of the structural region shown in (**b**). The interface of the TbMn_6_Sn_6_/Au heterostructure is marked by a green dashed line.

To get further insight into this emergent superconducting phase, the structural and elemental mappings have been performed in a high-resolution scanning transmission electron microscopy (STEM) system. The high-angle annular dark-field STEM (HAADF STEM) image of one typical TbMn_6_Sn_6_ single crystal is shown in Fig. 4a, which manifests a regular atomic stacking sequence along the *c*-axis of TbMn_6_Sn_6_. Figure 4b shows a STEM image of a heterostructure made by depositing 10 nm Au film on the (001) surface of TbMn_6_Sn_6_, where the interface superconductivity emerges. The top dark region is the carbon shielding layer, which is deposited during the thin lamellae preparation for STEM to prevent Au film from being damaged by the gallium ion beam. The bright stripe region in the middle of the image is the 10 nm thick Au film. The region just below the gold film (roughly marked by the dashed yellow rectangle) does not exhibit long-range ordered crystalline structures, but shows polycrystalline TbMn_6_Sn_6_ structure (Supplementary Text II and Fig. 13). In this work, this region is referred to the degraded TbMn_6_Sn_6_ layer. In the region a little far from the interface, the regular atomic interlayer stacking structure of TbMn_6_Sn_6_ is detected. The element distribution of the TbMn_6_Sn_6_/Au heterostructure can be revealed by the elemental mappings from the energy-dispersive X-ray spectroscopy (EDS). The blue stripe of the Au element distribution shown in Fig. 4c is consistent with the Au film region in Fig. 4b. The Mn and Tb shown in Figs. 4d and e are distributed in both the degraded and crystalline TbMn_6_Sn_6_ region. In Fig. 4f, the Sn element is relatively deficient in the degraded region near the interface compared to the regions with the regular TbMn_6_Sn_6_ atomic lattice, excluding the possibility of superconducting tin filamentary.

To further reveal the origin of the degraded layer, we also conducted STEM studies on as-grown TbMn_6_Sn_6_ samples. The results show that the tin-deficient degraded layer already exists in the as-grown samples, indicating the degraded layer is naturally formed near the surface of TbMn_6_Sn_6_ (Supplementary Text II and Fig. 14).

## Discussion

In summary, we discovered emergent superconductivity at the interface of TbMn_6_Sn_6_/metal heterostructures formed by depositing the non-superconducting metallic thin films, such as the nonmagnetic Au and Ag, and ferromagnetic Ni, on the surfaces of ferrimagnetic TbMn_6_Sn_6_. The superconductivity is also evidenced at PC interfaces between TbMn_6_Sn_6_ and non-superconducting metallic tips, including ferromagnetic Ni tip. We found that the emergent superconductivity at the interface is quasi-2D and couples to the magnetization inherited from the magnetic order of TbMn_6_Sn_6_ and exhibits hysteretic magnetoresistance. These findings suggest the emergence of a quasi-2D time-reversal-symmetry-breaking superconducting state at the interface of TbMn_6_Sn_6_/metal heterostructures.

**Fig. 5:**
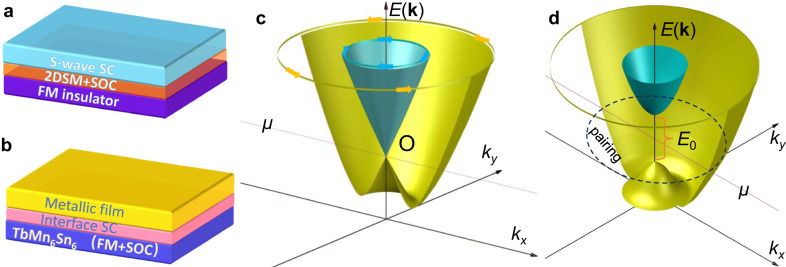
The theoretical scenario for the interface superconductivity of TbMn_6_Sn_6_/metal heterostructure. **a** The strategy of the proximity-induced topological superconductivity in stacked layers formed by an *s*-wave superconductor (SC), a 2D semiconductor (2DSM) with Rashba spin-orbit coupling (SOC), and a ferromagnetic (FM) insulator. **b** The schematic of the detected interface superconductivity in TbMn_6_Sn_6_/ metal heterostructures. **c** The schematic of the electron band structure *E*(**k**) of the 2D degraded layer with Rashba SOC without considering the exchange field from TbMn_6_Sn_6_. The opposite spin orientations along the effective Rashba SOC field direction are denoted by yellow and cyan arrows. The band with a yellow (cyan) color indicates this band possesses the same spin texture denoted by the yellow (cyan) arrows. Chemical potential *μ* is marked by a purple line. **d** The schematic of energy dispersion of the 2D degraded layer with Rashba SOC coupled to the kagome magnet TbMn_6_Sn_6_. *E*_0_ is the Zeeman gap. The dashed circle marked by ‘pairing’ indicates the Cooper pairing in the outer band when the chemical potential *μ* is located in the gap *E*_0_.

A plausible mechanism is that the carrier doping may take place near the interface and enable an emergent quasi-2D superconductor. Furthermore, near the interface, polycrystalline TbMn_6_Sn_6_ structures are revealed in the degraded layer by structural analyses (Supplementary Text II and Fig. 13). Consequently, the magnetization and strong spin-orbit coupling (SOC) from polycrystalline TbMn_6_Sn_6_ are expected to be inherited by the emergent interface superconductivity. As a result, the interface of the TbMn_6_Sn_6_/metal heterostructure contains, surprisingly, all the essential ingredients to generate chiral topological superconductivity, as proposed for 2D Rashba SOC semiconductors proximity coupled to an *s*-wave superconductor and a ferromagnetic insulator^26,27^, as illustrated in Fig. 5a. However, the theoretical proposal has not been realized presumably because the difficulty to meet the condition that proximity-induced superconductivity and ferromagnetism are coupled to each other in the semiconductor layer. These challenges are overcome in the TbMn_6_Sn_6_/metal heterostructures, since the quasi-2D superconductivity coupled with the magnetization and strong SOC are all established at the interface (Fig. 5b), thus providing a more realistic material platform for realizing chiral topological superconductivity. More specifically, the Rashba SOC leads to a pair of spin nondegenerate bands in the *k*_x_-*k*_y_ plane (Fig. 5c). The exchange field from the degraded TbMn_6_Sn_6_ layer opens a Zeeman energy gap (*E*_0_) at the crossing point of these two bands at *k*_x_ *= k*_y_ = 0 as shown in Fig. 5d. The emergent interface superconductivity opens up a superconducting gap Δ in the outer band if the chemical potential *μ* is located in the gap *E*_0_. When the condition *E*_0_ > (*μ*^2^ + Δ^2^)^1/2^ is satisfied, the proposed effective chiral *p*-wave superconducting state^26,27^ can be realized at the interface of the TbMn_6_Sn_6_/metal heterostructures. We note in passing that although the direct evidence has not been found at the current stage, if the interface superconductivity takes place in the kagome layers, topological superconductivity can also arise, which is discussed using a theoretical model in the Supplementary Text III and IV for different effective exchange couplings. Our findings provide a new direction for studying the interplay of ferromagnetism and superconductivity at the interface of kagome magnet and metal heterostructures, and at the same time, uncover a material platform to pursue time-reversal symmetry-breaking topological superconductivity.

## Methods

### Electrical transport and magnetization measurements

The electrical transport measurements of longitudinal and Hall resistance were performed in a commercial physical property measurement system (PPMS, Quantum Design) by using the standard four-electrode method. Vibrating sample magnetometer (VSM) magnetization measurements were carried out in a commercial magnetic property measurement system (MPMS3, Quantum Design).

### Cryogenic point contact measurements

The cryogenic point contact system contains the low-temperature platform and the PC system. The point contact is performed on the top stage of three-axis piezo stacks from Attocube by pressing sharp metallic tips onto the TbMn_6_Sn_6_ sample’s selected surface. The point-contact spectra are measured by using the standard lock-in method. The cryogenic circumstance is created by inserting the piezo stacks into the Leiden dilution refrigerator (CF450) with three-axis superconducting magnets (1 T/1 T/3 T). The lowest temperature used for the point contact experiments is 1.1 K in this work. More discussions of the point-contact Andreev-reflection spectroscopy can be found in Supplementary Text V in the Supplementary Information.

### Single-crystal growth

High-quality single crystals of TbMn_6_Sn_6_ were prepared by using a self-tin-flux growth method. Terbium ingots, manganese pieces, and tin lumps with a molar ratio of 1:6:20 were packed into an alumina crucible blocked with a piece of quartz wool on the top, which was then sealed in a fused quartz ampoule under vacuum. The ampoule was heated to 1000 °C and kept for a few hours, then slowly cooled down to the centrifuging temperature of 600 °C. While hot, the ampoule was inverted and spun in a centrifuge, forcing the excess tin into the quartz wool located in the crucible. This method produced several hexagonal flat single crystals of millimeters in size. To ensure a clean surface, some samples are mechanically polished before measurements. For some large crystals with a size up to 2 mm × 2 mm × 0.5 mm, the fresh (001) surfaces are obtained by a mechanically cleaving process. All the clean side surfaces are obtained by the polishing process.

### Scanning transmission electron microscopy (STEM) measurements

All the STEM samples were prepared by the focused ion beam (FIB, Helios G4) system. All samples were protected by depositing a 10 nm carbon layer in the LEICAEM ACE200 coating system before transferring them to the FIB system. Before the FIB treatment, a 2 μm carbon protection layer was further deposited under 30 kV, 0.1 nA experimental condition in the FIB system. Then, the samples were thinned using an accelerating voltage of 30 kV with a decreasing current from 240 to 50 pA, followed by a fine polish with an accelerating voltage of 2 kV with a current of 20 pA to reduce surface damage. A Titan Cubed Themis G2 double Cs-corrected scanning transmission electron microscope was used at 300 kV to obtain the HAADF STEM images and energy-dispersive X-ray spectroscopy (EDS) mappings with a probe-forming semi-angle of 30 mrad. The HAADF STEM images were acquired with a beam current of ~50 pA and a collection semi-angle snap in the range of 39–200 mrad, 145 mm camera length.

### Sample treatment and film deposition

Before preparing the TbMn_6_Sn_6_/metallic film heterostructures, pre-treating processes were applied to the TbMn_6_Sn_6_ samples, including mechanically cleaving (Fig. 2a, b), surface polishing (Fig. 2c, d, Supplementary Figs. 2 and 5), or only ultrasonic cleaning by ethanol and acetone (Fig. 2e–f, Supplementary Figs. 4, 15 and 20). The TbMn_6_Sn_6_ samples used in TbMn_6_Sn_6_/Au heterostructure s7 and s8 are obtained by mechanically cleaving one TbMn_6_Sn_6_ crystal into two parts. Subsequently, the samples were transferred to the e-beam evaporation chamber of an LJUHV E-400L E-Beam Evaporator. The 10 nm-thick Au (or 8 nm-thick Ni) films were deposited onto the sample surface at room temperature by the standard e-beam evaporation method with the electron energy of 8 keV. The chamber pressure is lower than 2 × 10^−6 ^Torr before deposition and was maintained below 4 × 10^−6 ^Torr during the evaporation process. The purities of the commercial Au and Ni targets are 99.999%, and a deposition rate of 0.5 Å/s (0.3 Å/s) was achieved by focusing the electron beam on the Au (Ni) target. The fabrication processes of heterostructures s7 and s8 are identical. The 10-nm thick Ag film in Supplementary Fig. 6 was grown at room temperature by using the direct current (DC) reactive magnetron sputtering method with the MSP-3200 Magnetron Sputtering System. In the sputtering chamber, the Ag film (target impurity, 99.99%) was deposited onto the TbMn_6_Sn_6_ surface at a rate of 2.7 Å/s with an Ar gas pressure of 0.9 Pa.

### Supplementary information

Supplementary Information

Peer Review File

## Data Availability

All data needed to evaluate the conclusions in the study are present in the paper and/or the Supplementary Information. The data that support the findings of this study are available from the corresponding authors upon request.

## References

[1] Reyren N (2007). Superconducting interfaces between insulating oxides. Science.

[2] Fu L, Kane CL (2008). Superconducting proximity effect and Majorana fermions at the surface of a topological insulator. Phys. Rev. Lett..

[3] Gozar A (2008). High-temperature interface superconductivity between metallic and insulating copper oxides. Nature.

[4] Wang Q (2012). Interface-induced high-temperature superconductivity in single unit-cell FeSe films on SrTiO_3_. Chin. Phys. Lett..

[5] Zhang W (2014). Direct observation of high-temperature superconductivity in one-unit-cell FeSe films. Chin. Phys. Lett..

[6] Xu J (2015). Experimental detection of a Majorana mode in the core of a magnetic vortex inside a topological insulator-superconductor Bi_2_Te_3_/NbSe_2_ heterostructure. Phys. Rev. Lett..

[7] Nadj-Perge, S (2014). Observation of Majorana fermions in ferromagnetic atomic chains on a superconductor. Science.

[8] Wang H (2016). Observation of superconductivity induced by a point contact on 3D Dirac semimetal Cd_3_As_2_ crystals. Nat. Mat..

[9] Wang H (2017). Discovery of tip induced unconventional superconductivity on Weyl semimetal. Sci. Bull..

[10] Cao Y (2018). Unconventional superconductivity in magic-angle graphene superlattices. Nature.

[11] Zhu W (2020). Interfacial superconductivity on the topological semimetal tungsten carbide induced by metal deposition. Adv. Mater..

[12] Klitzing KV, Dorda G, Pepper M (1980). New method for high-accuracy determination of the fine-structure constant based on quantized Hall resistance. Phys. Rev. Lett..

[13] Nayak C, Simon SH, Stern A, Freedman M, Sarma SD (2008). Non-Abelian anyons and topological quantum computation. Rev. Mod. Phys..

[14] Hasan MZ, Kane CL (2010). Colloquium: Topological insulators. Rev. Mod. Phys..

[15] Qi XL, Zhang S-C (2011). Topological insulators and superconductors. Rev. Mod. Phys..

[16] Elliott SR, Franz M (2015). Colloquium: Majorana fermions in nuclear, particle, and solid-state physics. Rev. Mod. Phys..

[17] Sato M, Ando Y (2017). Topological superconductors: a review. Rep. Prog. Phys..

[18] Yin J (2020). Quantum-limit Chern topological magnetism in TbMn_6_Sn_6_. Nature.

[19] Tanaka Y, Golubov AA (2007). Theory of the proximity effect in junctions with unconventional superconductor. Phys. Rev. Lett..

[20] Buzdin AI (2005). Proximity effects in superconductor/ferromagnet heterostructure. Rev. Mod. Phys..

[21] Jacob L, Alexander VB (2019). Odd-frequency superconductivity. Rev. Mod. Phys..

[22] Volkov AF, Bergeret FS, Efetov KB (2003). Odd triplet superconductivity in superconductor-ferromagnet multilayered structures. Phys. Rev. Lett..

[23] Schnyder AP, Ryu S, Furusaki A, Ludwig AWW (2008). Classification of topological insulators and superconductors in three spatial dimensions. Phys. Rev. B.

[24] Qi XL, Hughes TL, Raghu S, Zhang S-C (2009). Time-reversal-invariant topological superconductors and superfluids in two and three dimensions. Phys. Rev. Lett..

[25] Lian B, Sun X, Vaezi A, Qi XL, Zhang S-C (2018). Topological quantum computation based on chiral Majorana fermions. Proc. Natl Acad. Sci. USA.

[26] Sau JD, Lutchyn RM, Tewari S, Sarma SD (2010). Generic new platform for topological quantum computation using semiconductor heterostructures. Phys. Rev. Lett..

[27] Alicea J (2010). Majorana fermions in a tunable semiconductor device. Phys. Rev. B.

[28] Venturini G, ElIdrissi BC, Malaman B (1991). Magnetic properties of RMn_6_Sn_6_ (R = Sc, Y, Gd–Tm, Lu) compounds with HfFe_6_Ge_6_ type structure. J. Magn. Magn. Mater..

[29] Aoki D (2001). Coexistence of superconductivity and ferromagnetism in URhGe. Nature.

[30] Tinkham, M. Introduction to superconductivity. (Courier Corporation, 2004).

[31] Harper FE, Tinkham M (1968). The mixed state in superconducting thin films. Phys. Rev..

[32] Blonder GE, Tinkham M, Klapwijk TM (1982). Transition from metallic to tunneling regimes in superconducting microconstructions: excess current, charge imbalance, and supercurrent conversion. Phys. Rev. B.

[33] Wang H, Ma L, Wang J (2018). Tip-induced or enhanced superconductivity: a way to detect topological superconductivity. Sci. Bull..

[34] Wang H (2020). Ferromagnetic tip induced unconventional superconductivity in Weyl semimetal. Sci. Bull..

