# Supplementary material for: Emergent superconductivity in topological-kagome-magnet/metal heterostructures
Source: Nat Commun. 2023 Nov 2;14:6998. doi: 10.1038/s41467-023-42779-1 (PMC10622413; doi:10.1038/s41467-023-42779-1)
Supplement: Supplementary file 1 — Supplementary Information [file 41467_2023_42779_MOESM1_ESM.pdf]

Supplementary Information for

Emergent superconductivity in topological-kagome-magnet/metal heterostructures

He Wang[1,2,8], Yanzhao Liu[1,8], Ming Gong[1,8], Hua Jiang[3,8], Xiaoyue Gao[1], Wenlong Ma[1], Jiawei Luo[1], Haoran Ji[1], Jun Ge[1], Shuang Jia[1], Peng Gao[1], Ziqiang Wang[4]✉, X. C. Xie[1,5,6] & Jian Wang[1,5,7]✉

[1]International Center for Quantum Materials, School of Physics, Peking University, Beijing 100871, China.

[2]Center for Quantum Physics and Intelligent Sciences, Department of Physics, Capital Normal University, Beijing 100048, China.

[3]Institute for Advanced Study, Soochow University, Suzhou 215006, China.

[4]Department of Physics, Boston College, Chestnut Hill, MA 02467, USA.

[5]Hefei National Laboratory, Hefei 230088, China.

[6]Institute for Nanoelectronic Devices and Quantum Computing, Fudan University, Shanghai 200433, China.

[7]Collaborative Innovation Center of Quantum Matter, Beijing 100871, China.

[8] These authors contributed equally: He Wang, Yanzhao Liu, Ming Gong and Hua Jiang.

✉**email: jianwangphysics@pku.edu.cn (Jian Wang), wangzi@bc.edu (Ziqiang Wang)**

Supplementary Text I

Similar to the $TbMn_6Sn_6$/Ni film heterostructure (Supplementary Fig. 2), the superconductivity is also successfully induced by using ferromagnetic Ni tips for point contact (PC) on $TbMn_6Sn_6$. We present a typical set of the PC results obtained on the (001) surface of $TbMn_6Sn_6$ using the Ni tip in Supplementary Fig. 9. The temperature dependence of the normalized PC resistance (R/R_{4K}) in Supplementary Fig. 9a illustrates that the T_c of the induced-superconducting state is about 3.45 K. Supplementary Fig. 9b and 9c show the temperature and the magnetic field evolutions of the point contact spectra (PCS). There is a notable conductance enhancement at low bias in the PCS at $T< T_C$. This enhancement is gradually suppressed by increasing temperature or magnetic fields, confirming the observation of superconductivity. The MR curves at 1.3 K (see Supplementary Fig. 9d) show a clear hysteresis loop when the magnetic field is swept along the *c*-axis of $TbMn_6Sn_6$. The minima of MR curves are located at (+/-) 0.035 T when the field is ramped from (-/+) 3 T to (+/-) 3 T (Supplementary Fig. 9d), similar to the hysteresis results for $TbMn_6Sn_6$/PtIr tip junction (Fig. 3d). The superconductivity is suppressed around 1.0 T. Related PC results measured using the Ni tip on the side surface of $TbMn_6Sn_6$ are shown in Supplementary Fig. 10.

Supplementary Text II

We further analyzed the lattice structures in the degraded layer of the $TbMn_6Sn_6$/Au heterostructure. In the degraded layers of two typical $TbMn_6Sn_6$/Au heterostructures s8 (shown in Fig. 4 in the main text) and s17, we can identify some regions showing lattice structures that are consistent with the crystal structure of $TbMn_6Sn_6$. Supplementary Fig. 13 displays three high-angle annular dark-field STEM (HAADF STEM) images of the degraded layers in s8 (Supplementary Fig. 13a) and s17 (Supplementary Fig. 13b-c). These images present regions showing crystal structures in the degraded layer, and the interplanar lattice spacing values in these regions are approximately 4.5 Å. Using the PDF Card (NO. 01-072-3490) as a reference, this value corresponds to the space distance of (002) $TbMn_6Sn_6$ along the [001] crystal orientation (4.502 Å). These findings suggest that the degraded layer mainly consists of the polycrystalline $TbMn_6Sn_6$.

To further reveal the origin of degraded layers, we also conducted STEM studies on as-grown $TbMn_6Sn_6$ samples, which are shown in Supplementary Fig. 14. Similar to the TEM data of $TbMn_6Sn_6$/Au heterostructure shown in Fig. 4 in the main text, the TEM image of the as-grown $TbMn_6Sn_6$ also shows the degraded layer exists at the sample surface. Furthermore, the deficiency of the Sn element is also observed in the degraded region near the surface of the as-grown $TbMn_6Sn_6$ (Supplementary Fig. 14d). Therefore, the degraded layer already exists before the metal film (Au, Ag, and Ni) deposition or metallic tip contact measurements, which explains why both metal thin film/$TbMn_6Sn_6$ and point-contact/$TbMn_6Sn_6$ show similar interface superconductivity.

Supplementary Text III

The surface superconductivity with *s*-wave pairing symmetry in the quantum-limit Chern topological magnet $TbMn_6Sn_6$[1] induced by a strong Rashba type SOC is proposed to explain the observed interface superconductivity, considering that the superconductivity takes place in the Kagome layer with the strong exchange coupling. The interplay between the topological band structure on the (001) surface and the *s*-wave pairing may lead to *p*-wave-like topological superconductivity. This Rashba-type SOC originates from the electric field induced by the strong structural inversion asymmetry on the surface[2]. Experiments and numerical simulations have proved the large Rashba SOC on the surface of Au[3,4] as well as the interface between ferro- and non-magnetic materials[5]. The effective Hamiltonian[1] of the system with Rashba type SOC can be written as $\boldsymbol{H} = -\sum_{<i,j>} \boldsymbol{t c_i^{+} c_j} - (\boldsymbol{\mu_0} + \boldsymbol{\mu}) \sum_i \boldsymbol{c_i^{+} c_i} - \boldsymbol{J} \sum_i \boldsymbol{c_i^{+} \sigma_z c_i} + \boldsymbol{i\lambda_{K-M}} \sum_{i,j} \boldsymbol{v_{ij} c_i^{+} \sigma_z c_j} + \boldsymbol{i\lambda_R} \sum_{i,j} \boldsymbol{c_i^{+}} \left(\boldsymbol{\sigma} \times \boldsymbol{b_{ij}}\right) \cdot \boldsymbol{z c_j}$, with hopping $\mathbf{t}$ and chemical potential $\boldsymbol{\mu}$. $\boldsymbol{J}$ is the effective exchange coupling strength. $\boldsymbol{v_{ij}} = \mathbf{2}/\sqrt{\mathbf{3}}(\boldsymbol{b_i} \times \boldsymbol{b_j}) \cdot \boldsymbol{z}$ and $\boldsymbol{\lambda_{K-M}}$ are the parameters of the Kane-Mele type SOC. $\boldsymbol{b_{ij}}$ represent the bond unit vectors that connect neighbor sites i and j (Supplementary Fig. 16a). $\boldsymbol{\lambda_R}$ is the coupling strength of the Rashba-type SOC.

Hereinbelow, we set t = 1, J = 5, $\mu_0 = -J - t$ and λ_{K-M} = 0.1 with the arbitrary unit. When *s*-wave superconductivity is induced, the superconducting pairing parameter Δ = 1. In the absence of superconductivity and Rashba-type SOC, the system is in a quantum anomalous Hall state, and chiral edge states are present (Supplementary Fig. 16b). Since the system is spin-polarized, the formation of conventional *s*-wave superconductivity is hampered. When Rashba-type SOC is introduced, the spin-up and spin-down components of the electrons are mixed. The formation of conventional *s*-wave superconductivity becomes possible and leads to a pairing term to the total Hamiltonian: $\mathcal{H} = H + H_{s-wave}$, where $H_{s-wave} = \Delta \sum_i c_{i\uparrow}^{+} c_{i\downarrow}^{+} + \Delta^* \sum_i c_{i\downarrow} c_{i\uparrow}$. By calculating the BdG Chern number N[6,7], we summarize the phase diagram in terms of μ and λ_R in Supplementary Fig. 16c. The system can enter into the chiral topological superconducting phase with N = 1. We take a typical value $\mu = -2$ and increase λ_R, the BdG band is shown in Supplementary Fig. 16e and 16f, where a pair of chiral Majorana modes emerge around E = 0. Furthermore, as shown in Supplementary Fig. 16d, the effective superconducting gap Δ_{eff} is enhanced by increasing λ_R and can be suppressed by the finite temperature in real experimental conditions. Therefore, a large Rashba SOC is necessary to host the superconductivity with the BdG Chern number N = 1 (ref. 7).

Supplementary Text IV

As discussed in Supplementary Text II, the $TbMn_6Sn_6$ sample near the interface has a naturally formed degraded layer possessing polycrystalline $TbMn_6Sn_6$. When we consider the Kagome structure in the degraded $TbMn_6Sn_6$ layer, the interplay between the topological band structure on the (001) surface and the *s*-wave pairing could also lead to effective *p*-wave topological superconductivity. This Rashba-type SOC originates from the dramatic structural inversion asymmetry on the degraded layer shown in Fig. 4 (ref. 2). The effective Hamiltonian[1] of the system with Rashba-type SOC can be written as $\boldsymbol{H} = -\sum_{<i,j>} \boldsymbol{t c_i^+ c_j} - (\boldsymbol{\mu_0} + \boldsymbol{\mu}) \sum_i \boldsymbol{c_i^+ c_i} - \boldsymbol{J} \sum_i \boldsymbol{c_i^+ \sigma_z c_i} + \boldsymbol{i\lambda_{K-M}} \sum_{i,j} \boldsymbol{v_{ij} c_i^+ \sigma_z c_j} + \boldsymbol{i\lambda_R} \sum_{i,j} \boldsymbol{c_i^+} \left(\boldsymbol{\sigma} \times \boldsymbol{b_{ij}}\right) \cdot \boldsymbol{z c_j}$, with hopping $\mathbf{t}$ and chemical potential $\boldsymbol{\mu}$. $\boldsymbol{J}$ is the effective exchange coupling strength. $\boldsymbol{v_{ij}} = \mathbf{2}/\sqrt{\mathbf{3}}(\boldsymbol{b_i} \times \boldsymbol{b_j}) \cdot \boldsymbol{z}$ and $\boldsymbol{\lambda_{K-M}}$ are the parameters of the Kane-Mele type SOC. $\boldsymbol{b_{ij}}$ represent the bond unit vectors that connect neighbor sites i and j (Supplementary Fig. 17a). $\boldsymbol{\lambda_R}$ is the coupling strength of the Rashba-type SOC.

Hereinbelow, considering the weaker effective exchange coupling strength in the degraded interface layer, we set t = 1, J = 0.75, $\mu_0 = -J - t$ and λ_{K-M} = 0.1 with the arbitrary unit. When *s*-wave superconductivity is induced, the superconducting pairing parameter Δ=0.5. In the absence of superconductivity and Rashba-type SOC, the system is in a quantum anomalous Hall state. Since the system is spin-polarized due to the exchange coupling, the formation of conventional *s*-wave superconductivity is hampered. When Rashba-type SOC is introduced, the spin-up and spin-down components of the electrons are mixed as shown in Supplementary Fig. 17b. The formation of conventional *s*-wave superconductivity becomes possible and leads to a pairing term to the total Hamiltonian: $\mathcal{H} = H + H_{s-wave}$, where $H_{s-wave} = \Delta \sum_i c_{i\uparrow}^+ c_{i\downarrow}^+ + \Delta^* \sum_i c_{i\downarrow} c_{i\uparrow}$. By calculating the BdG Chern number N[6,7], we summarize the phase diagram in terms of μ and λ_R in Supplementary Fig. 17c. The system can enter into the chiral topological superconducting phase with N = 1 near $\mu = 3$, which lies in the original topological flat band of the lower magnetic sub-band. We take a typical value $\mu = 3.2$ and increase λ_R, the BdG band is shown in Supplementary Figs. 17e and 17f, where a pair of chiral Majorana modes emerge around E = 0. Furthermore, as shown in Supplementary Fig. 17d, the effective superconducting gap Δ_{eff} is enhanced by increasing λ_R and can be suppressed by the finite temperature in real experimental conditions. Therefore, a large Rashba SOC is necessary to host the superconductivity with the BdG Chern number N = 1 (ref. 7).

Supplementary Text V

The point-contact Andreev-reflection spectroscopy is a powerful tool for studying the properties of superconducting materials[8,9]. By employing the Andreev reflection process[10], which occurs when an electron in the normal metal is reflected as a hole at the contact interface between the superconductor and normal metal, the point-contact Andreev-reflection spectroscopy can be used to measure the superconducting gap, determine the superconducting order parameter, analyze the pairing symmetry, and understand the pairing mechanism[11-13]. The point contact with the size much smaller than the electron elastic mean free path, locates in the ballistic regime. For the size larger than the elastic mean free path, but smaller than the inelastic mean free path, the point contact locates in the intermediate regime[14]. When point-contact Andreev-reflection measurements are conducted on a conventional superconductor, there would be only two conductance peaks in the point-contact spectra for the case in the ballistic regime, and two conductance peaks combined with two conductance dips in the PCS for the case in the intermediate regime. In our experiments, we tried to fit PCS by using the Blonder, Tinkham, and Klapwijk (BTK)[15] model, a typical example is shown in Supplementary Fig. 12a. The fitted superconducting gap value is 0.66 meV, which is approximately equal to the value estimated by the bias voltage of the conductance peak (~0.70 meV). In Supplementary Fig. 12b, we plot the bias voltage of conductance peaks in PCS obtained by Ni tips versus T_c values. The T_c values are estimated from R-T curves of the corresponding superconducting state. As shown in Supplementary Fig. 12b, a positive correlation between peak bias and T_c values is detected, demonstrating the PCS results are valid.

Supplementary Text VI

In the PCS of three PC states (marked as PC1, PC2, and PC3) made by pressing the Au tip onto the (001) $TbMn_6Sn_6$ surface, zero-bias conductance peaks (ZBCPs) are observed (Supplementary Fig. 18). As shown in Supplementary Fig. 18a, the height of ZBCP at 1.1 K and 0 T for PC1 is far greater than twice the conductance at high bias, which is at odds with the conventional Andreev reflection[9]. To identify the origin of the ZBCPs, several mechanisms should be considered, including the Kondo effect[16], weak anti-localization[17,18], reflectionless tunneling[19], Andreev bound states[20,21,] and formation of Majorana zero modes[20,21]. Firstly, the ZBCP originating from the Kondo effect induced by magnetic impurities will split and move to finite energy by increasing the external magnetic field, which is obviously inconsistent with the non-splitting ZBCP under an external magnetic field in our experiments (Supplementary Fig. 18a). Secondly, the field scale of the reflectionless tunneling and weak anti-localization effect can be estimated by B_0 ~ (h/eA)[17], where A is the characteristic area of the electron trajectory perpendicular to the magnetic field. With the tunneling area radius ~ 1 μm in our PC measurements, the field scale of several milliTesla is obtained, which is much smaller than the magnetic field range where the ZBCPs exist in our experiments. Thirdly, the temperature dependence of the zero-bias conductance height shown in Supplementary Fig. 18c is fitted well by an exponential function rather than the 1/T behavior typically expected from the Andreev bound state[20,21]. The exponential temperature dependence of zero-bias conductance height and the FWHM of the ZBCP is consistent with the characteristic of the Majorana bound state[20,21] but not expected for the superconducting point contact in the thermal regime. Thus, the topological non-trivial superconductivity is a potential explanation for the ZBCP shown in Supplementary Fig. 18.

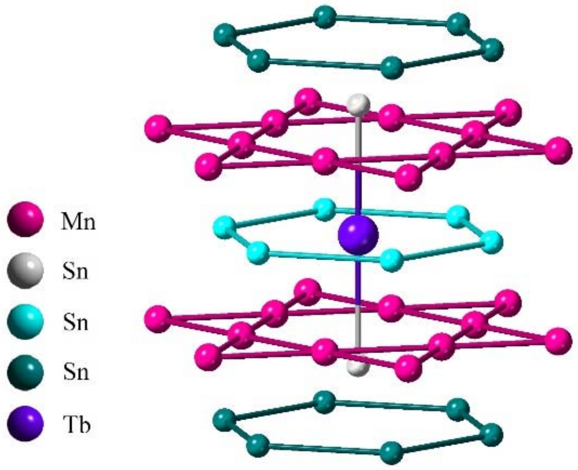

Supplementary Fig. 1 | The schematic of the crystal structure of $TbMn_6Sn_6$.

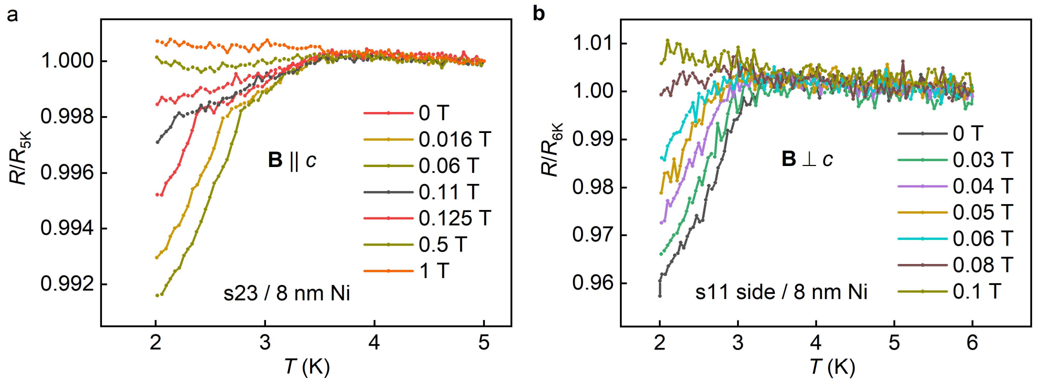

Supplementary Fig. 2 | a The normalized resistance versus temperature curves of $TbMn_6Sn_6$ (s23) capped 8 nm Ni on the (001) surface. The magnetic field is applied along the out-of-plane direction (**B**||*c* axis). **b** The normalized resistance versus *T* curves of $TbMn_6Sn_6$ (s11) with 8 nm Ni capped on the side surface. The magnetic field is applied perpendicular to the side surface (**B**⊥*c* axis).

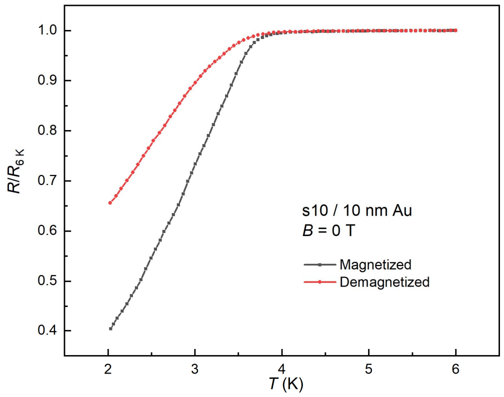

Supplementary Fig. 3 | The normalized resistance versus temperature curves of $TbMn_6Sn_6$ (s10) capped 10 nm Au on the (001) surface after magnetization and demagnetization treatment.

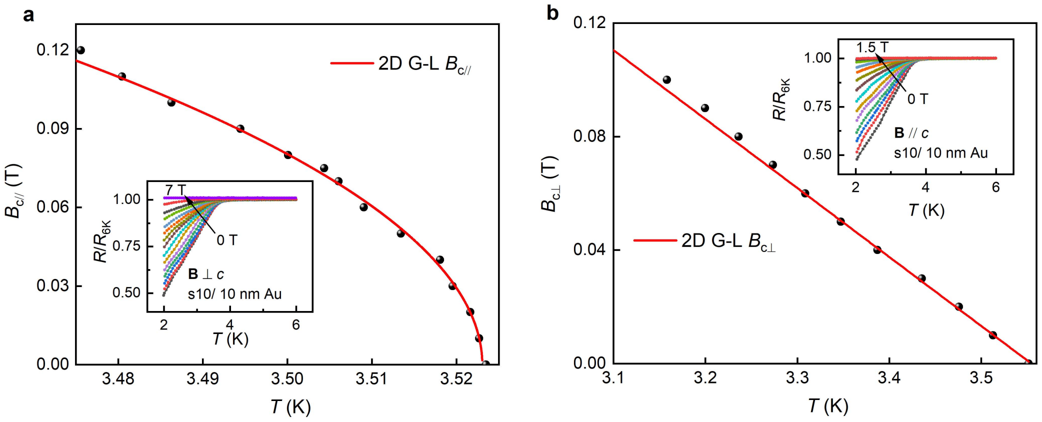

Supplementary Fig. 4 | 2D Ginzburg-Landau fitting of the temperature dependence of the critical magnetic field. a The temperature dependence of the critical magnetic field $B_{c//}(T)$ of a $TbMn_6Sn_6$ sample (s10) capped with 10 nm Au film under the magnetic field applied perpendicular to the *c*-axis near T_c. The red curve is the 2D G-L fitting, showing the $(T_c\text{-}T)^{1/2}$ dependence. The inset shows the temperature dependence of normalized resistance under different magnetic fields applied perpendicular to the *c*-axis. **b** The temperature dependence of the critical magnetic field $B_{c\perp}$ (T) of s10 capped with 10 nm Au film under the magnetic field applied along the *c*-axis near T_c. The red curve is the 2D G-L fitting, showing *T*-linear dependence. The inset shows the temperature dependence of normalized resistance under different magnetic fields applied along the *c*-axis. B_c is defined as the magnetic field corresponding to 95% normal resistance.

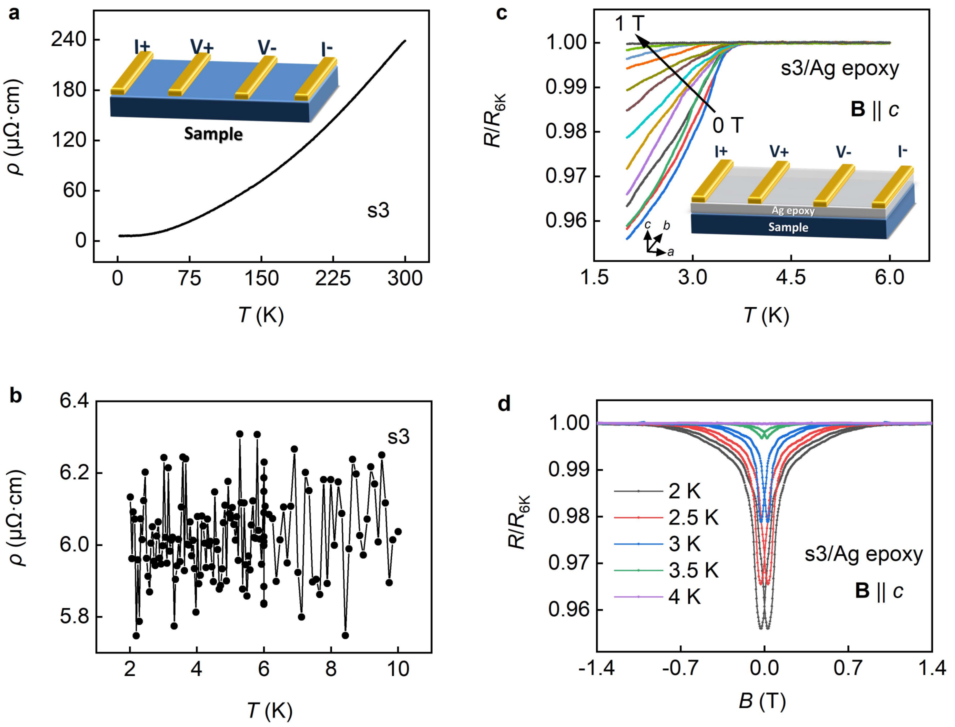

Supplementary Fig. 5 | The transport results of the $TbMn_6Sn_6$ (s3) capped with silver epoxy on the (001) surface. a Resistivity (ρ) versus temperature (T) curve of sample s3. Inset: the schematic of the standard four-electrode configuration. **b** The zoom-in of ρ-T curve below $T < 10$ K. **c** The evidence of superconductivity for Ag epoxy-coated (001) surface of $TbMn_6Sn_6$ (s3). Inset: the schematic of the standard four-probe measurements for the $TbMn_6Sn_6$/Ag epoxy sample. **d** The magnetoresistance curves of $TbMn_6Sn_6$ (s3) capped with silver epoxy on (001) surface at selected temperatures. The magnetic field in this figure is applied along the out-of-plane direction (**B**||c axis).

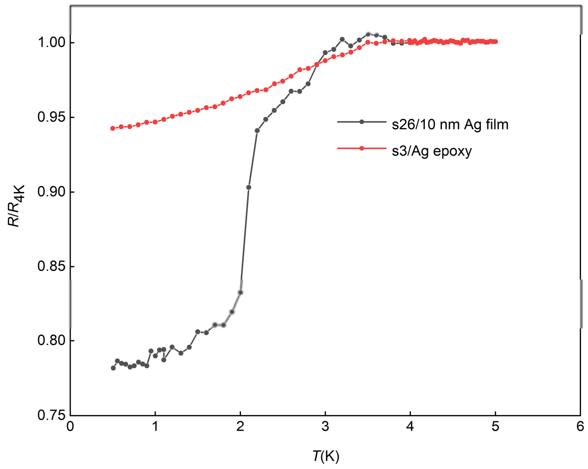

Supplementary Fig. 6 | The normalized resistance versus temperature curves of $TbMn_6Sn_6$ capped with 10 nm Ag film and silver epoxy on the (001) surface without an external magnetic field.

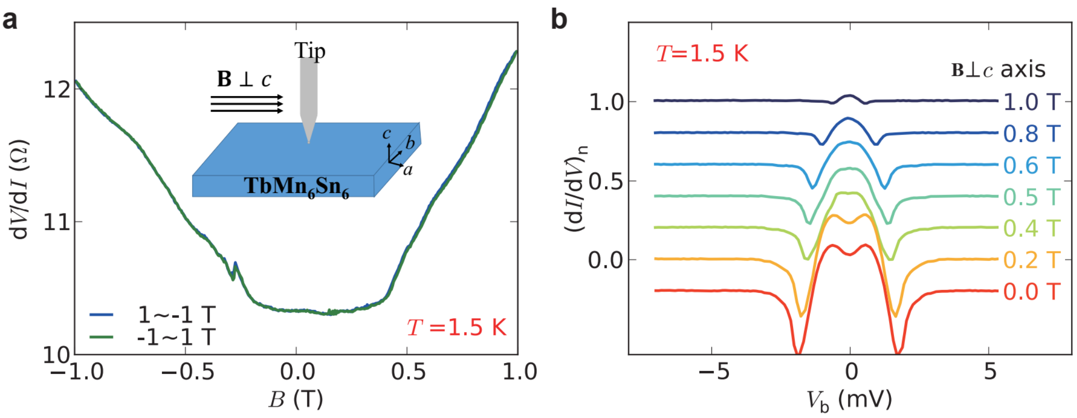

Supplementary Fig. 7 | The related results of the point-contact (PC) state shown in Fig. 3 in the main text. The point contact is formed by pressing the PtIr tip on the (001) surface of $TbMn_6Sn_6$. a The MR curves show no hysteresis when the magnetic field is applied at 1.5 K. Inset: the schematic of the PC configuration, the magnetic field is applied along the in-plane direction (**B**⊥*c* axis). **b** The normalized PCS at 1.5 K with different in-plane *B* values. The magnetic field is swept from 1 T to 0 T. The PCS at *B* = 1 T still presents superconducting features, indicating the superconducting critical field along the in-plane direction is larger than 1 T.

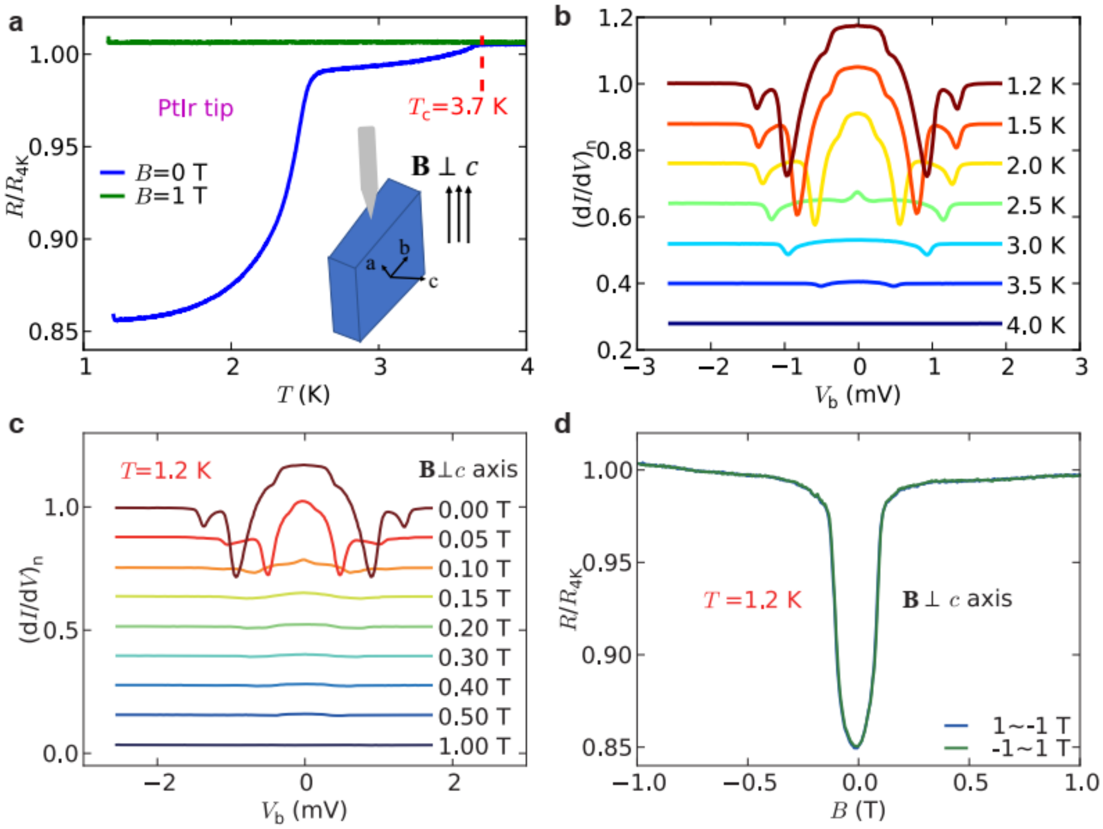

Supplementary Fig. 8 | The evidence of superconductivity at the point-contact (PC) interface formed by pressing PtIr tip onto the side surface of $TbMn_6Sn_6$. a The temperature dependence of the normalized PC resistance (R/R_{4K}) at zero bias without (blue curve) and with (green curve) applying the magnetic field. Inset: the magnetic field in **a**, **c**, and **d** is applied perpendicular to the side surface and the PC interface (**B**⊥*c* axis). **b** The temperature dependence of the normalized PCS. **c** The normalized PCS at different magnetic fields at 1.2 K, suggesting the critical magnetic field perpendicular to the superconducting layer is smaller than 1 T. The magnetic field is ramping from 0 T to 1 T. The PCS curves in **b** and **c** are shifted for clarity. **d** The MR measurements of the PC at 1.2 K. The PC resistance in the normal state is 4.0 Ω.

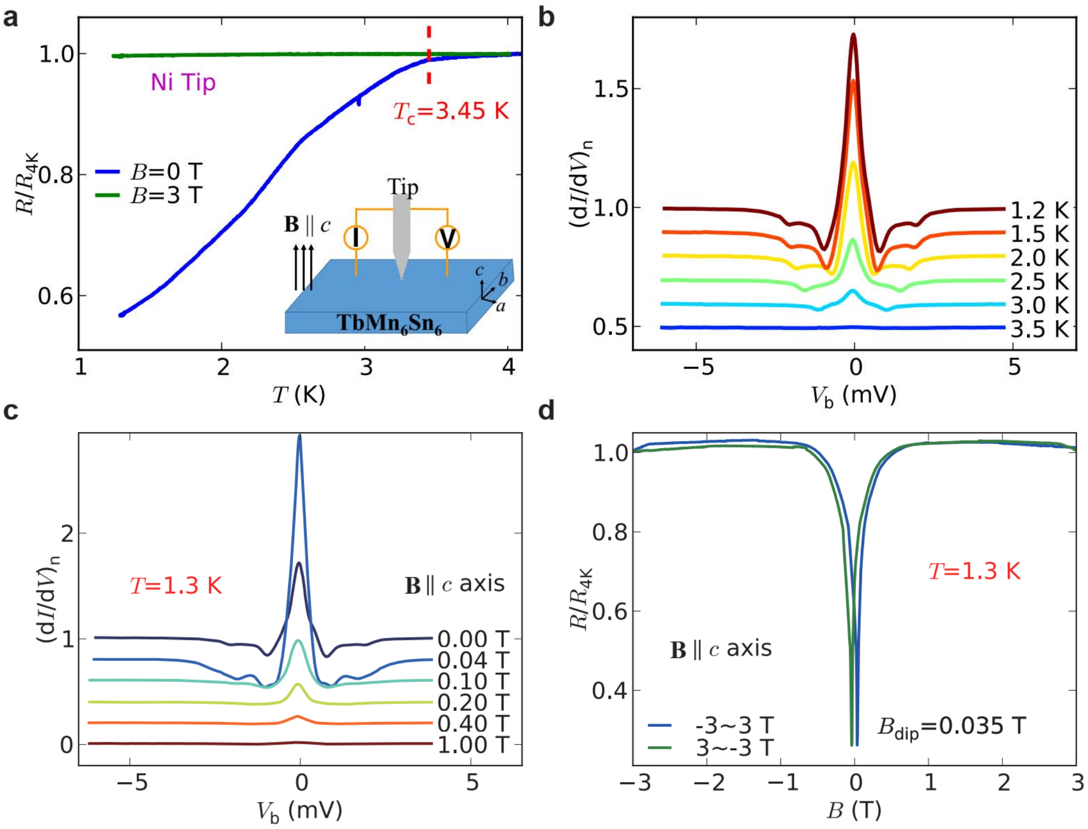

Supplementary Fig. 9 | The evidence of superconductivity at the point contact (PC) formed by pressing the ferromagnetic tip (Ni) onto the (001) surface of $TbMn_6Sn_6$. a The temperature dependence of the normalized zero-bias resistance of the PC without (blue curve) and with (green curve) applying the magnetic field. Inset: the schematic of the PC configuration, the magnetic field is applied along the out-of-plane direction (**B**||*c* axis). **b-c** The temperature and magnetic field dependence of the normalized PCS. The PCS curves are shifted for clarity. The related magnetic field is swept from 1 T to 0 T at 1.3 K. **d** The MR curves of the PC at 1.3 K. All the magnetic fields in this figure are applied along the out-of-plane direction (**B**||*c* axis). The PC resistance in the normal state is 15.3 Ω.

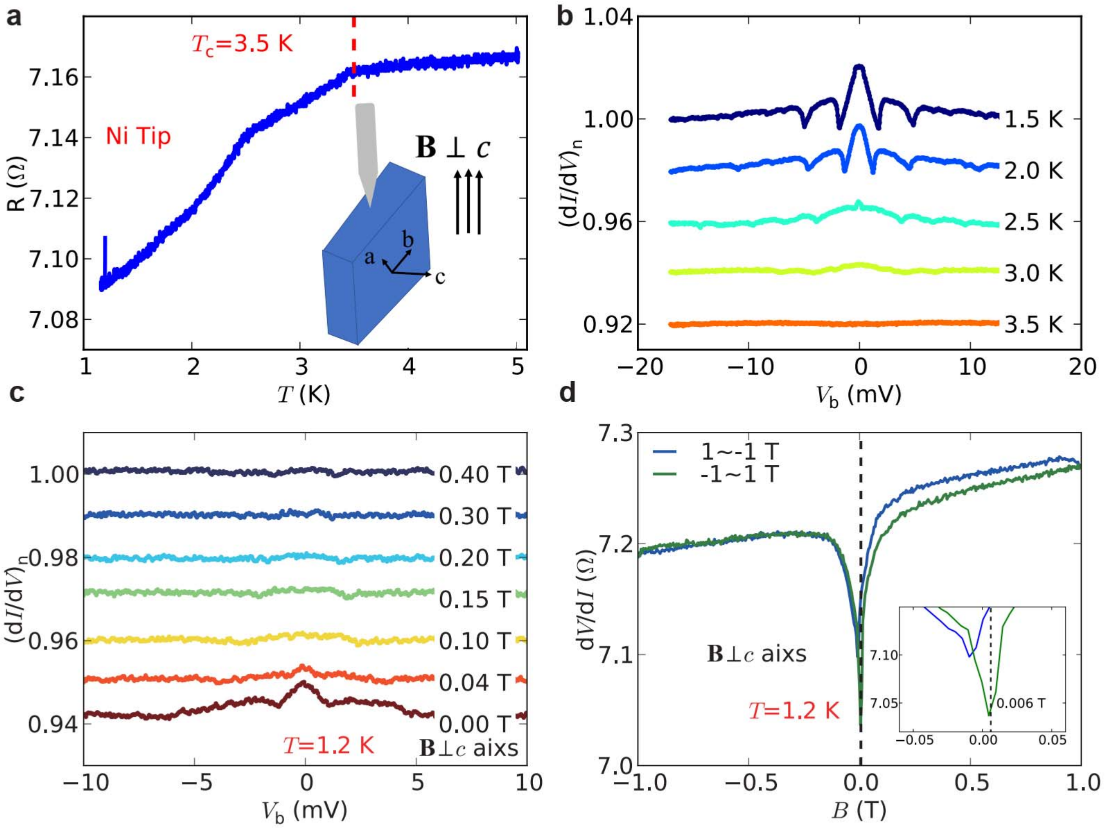

Supplementary Fig. 10 | The PC measurements by pressing a Ni tip to the side surface of $TbMn_6Sn_6$. a The *R*-*T* curve of PC, showing the T_c is about 3.5 K. Inset: the magnetic field in **c** and **d** is applied perpendicular to the side surface and the PC interface (**B**⊥*c* axis). **b**-**c** The temperature and magnetic field evolution of normalized PCS. The related magnetic field is ramping from 0.4 T to 0 T. **d** The MR curves show a hysteresis loop in the magnetic field perpendicular to the side surface (**B**⊥*c* axis). Inset: zoom-in of the MR dips. The conductance dips locate at ±0.006 T, indicating the loop is contributed by the Ni tips. The PC resistance in the normal state is 7.2 Ω.

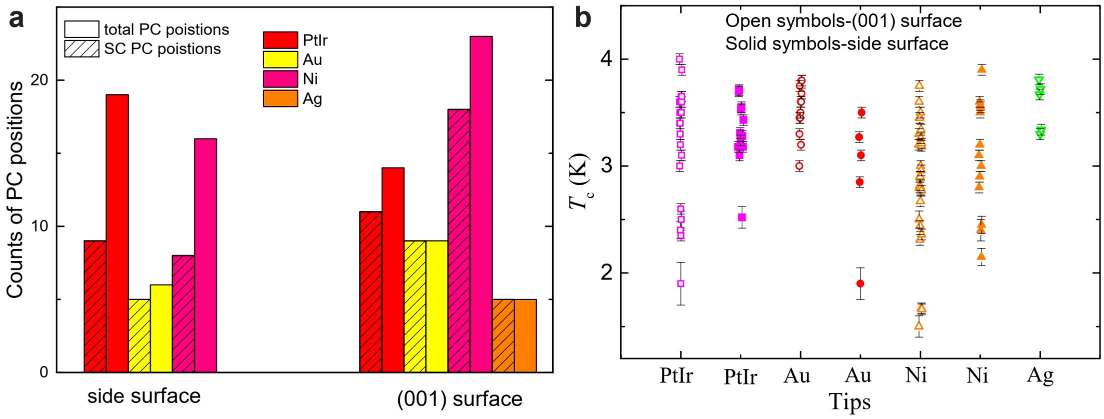

Supplementary Fig. 11 | a The statistics on the emergent superconductivity at the interface of the point contact made on either (001) surface or side surface using different kinds of tips. **b** The statistic chart of the T_c values of emergent superconducting states at the interface between $TbMn_6Sn_6$ and different metallic tips.

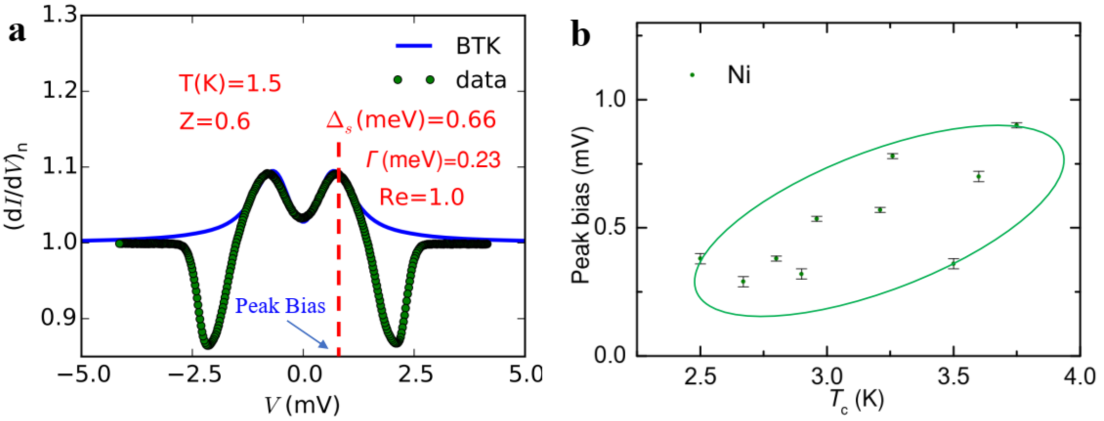

Supplementary Fig. 12 | The analysis of the conductance peaks of point-contact spectra (PCS). **a** The Blonder, Tinkham and Klapwijk (BTK)[15] model fitting of one typical normalized point-contact spectrum, where Z is the barrier parameter, Γ is the broadening parameter, Δ_s is the fitting gap value and Re is the extra normalized resistance. **b** The statistics of the conductance-peak bias voltages and the superconducting transition temperature T_c for the point contacts made on the (001) surface of $TbMn_6S_6$ by using Ni tips. A positive correlation between peak bias and T_c values is detected.

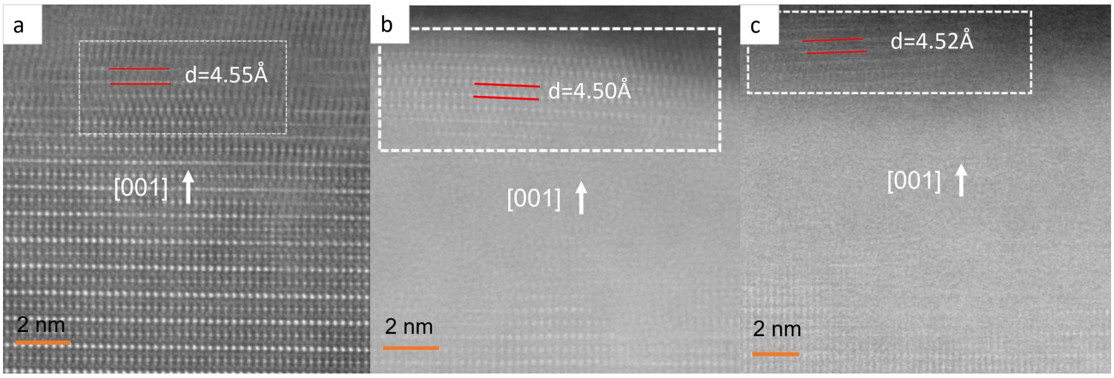

Supplementary Fig. 13 | Further analyses of the microstructure of the degraded $TbMn_6Sn_6$ layer near the interface of two typical $TbMn_6Sn_6$/Au heterostructures s8 (a) and s17 (b-c). The $TbMn_6Sn_6$/Au heterostructures are fabricated by depositing 10 nm Au film on the (001) surface of the $TbMn_6Sn_6$ single crystal. The selected area is marked by white squared frames. **a-c** Cross-sectional HAADF STEM images of the crystal lattice in the degraded $TbMn_6Sn_6$ layer near the interface of $TbMn_6Sn_6$/Au heterostructures s8 (**a**) and s17 (**b-c**). The interplanar spacing values of the lattices are marked by two red lines. The white arrows indicate the [001] orientation of the bulk $TbMn_6Sn_6$ single crystal. The *R*-*T* curves of s17 can be referred to Supplementary Fig. 20. The $TbMn_6Sn_6$ sample used in s8 is obtained by mechanically cleaving without a polishing process. The $TbMn_6Sn_6$ sample used in s17 is only ultrasonic cleaned by ethanol and acetone without surface polishing.

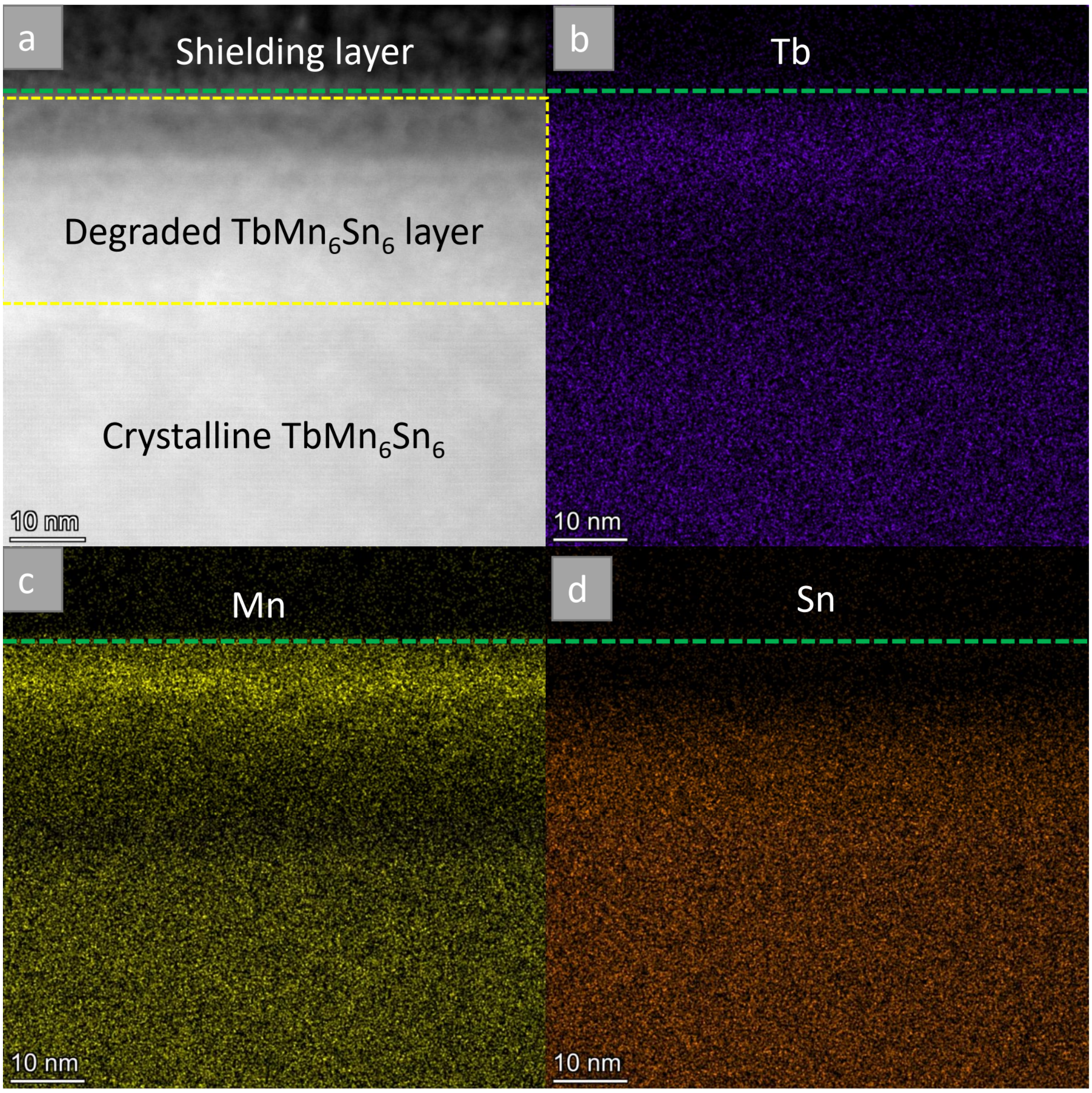

Supplementary Fig. 14 | Structural image and elemental mappings of the as-grown $TbMn_6Sn_6$ single crystal. a A cross-sectional HAADF STEM image of the $TbMn_6Sn_6$ single crystal surface. **b-d** The EDS mappings of Tb, Mn, and Sn elements distribution of the HAADF STEM image **a**. The interface between the $TbMn_6Sn_6$ and the shielding layer, a carbon-protecting layer with a thickness of more than 100 nm, is marked by a green dashed line. The degraded $TbMn_6Sn_6$ layer is marked by a yellow dashed line box.

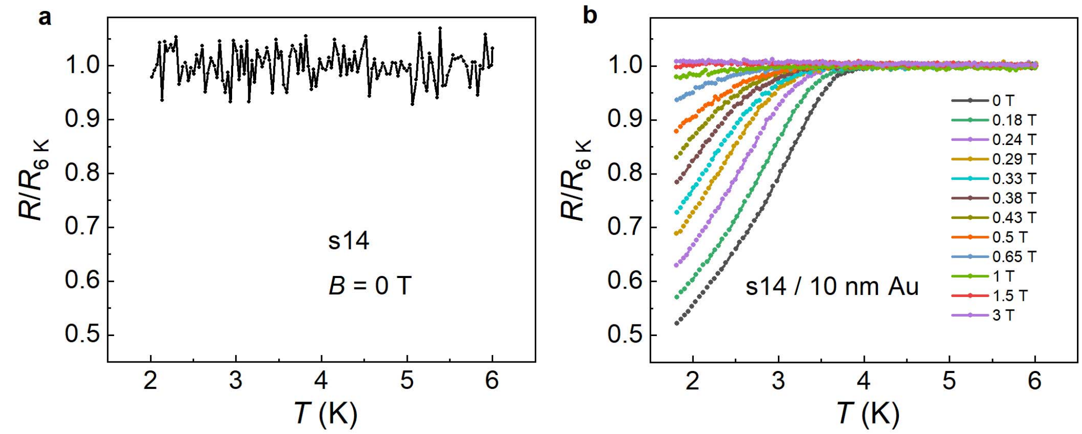

Supplementary Fig. 15 | The normalized resistance versus temperature curves of $TbMn_6Sn_6$ s14 (**a**) and $TbMn_6Sn_6$ s14 capped 10 nm Au on the (001) surface (**b**). The surface of s14 is only ultrasonic cleaned by ethanol and acetone to avoid the possible influence of mechanical force.

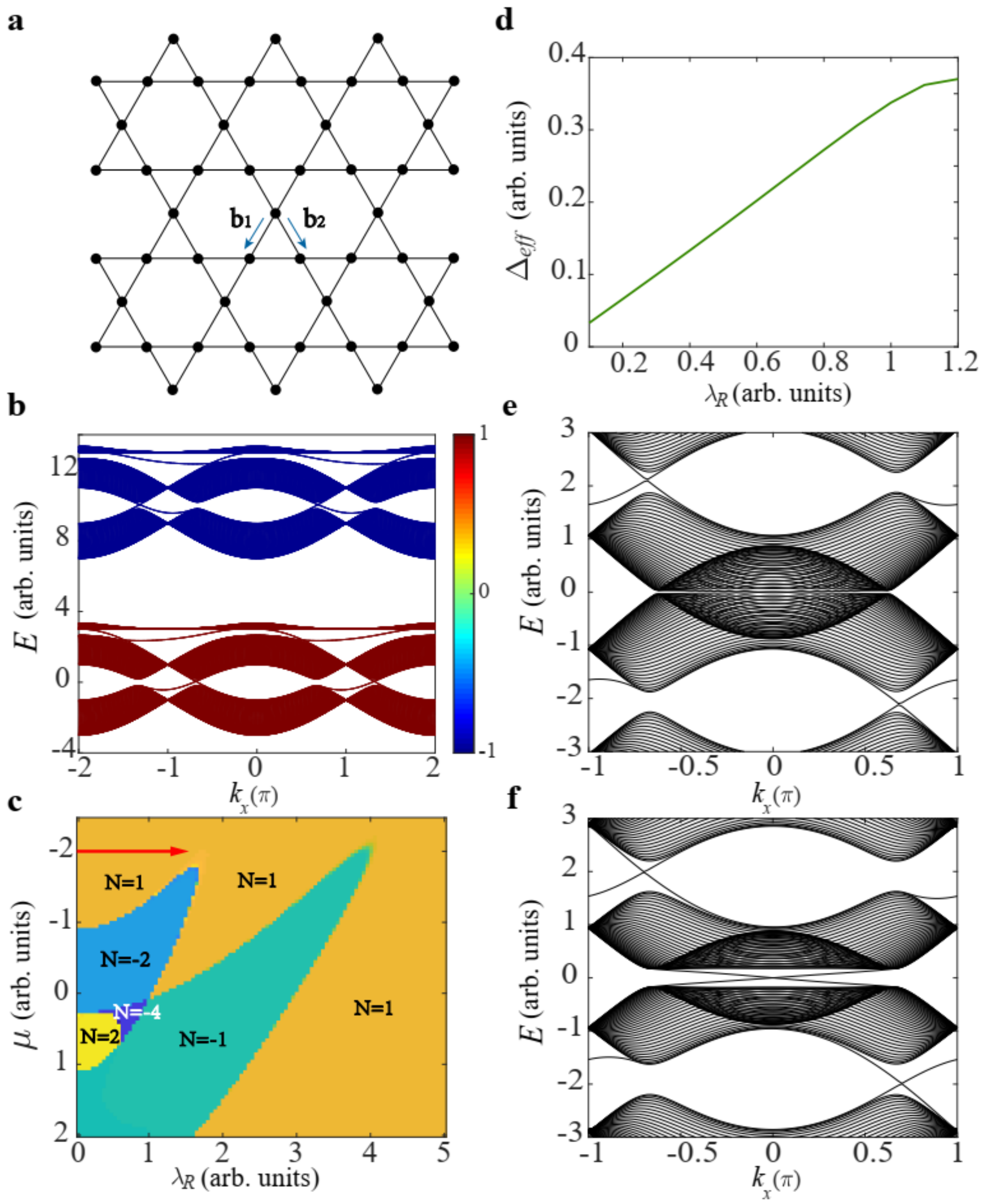

Supplementary Fig. 16 | Theoretical analyses of the emergent superconductivity of the Chern topological magnet/metal heterostructure, considering that the superconductivity takes place in the kagome layer with the strong exchange coupling. a Kagome lattice with unit vectors $\mathbf{b}_1$ and $\mathbf{b}_2$ connecting the nearest bonds in one unit cell. **b** Band structure on the (001) surface without superconductivity and Rashba-type SOC. The colormap represents the spin polarization along the z direction. **c** Phase diagram of the Bogoliubov-de Gennes (BdG) Chern number. The red arrow locates at $\boldsymbol{\mu} = -\mathbf{2}$. **d** Superconducting gap Δ_{eff} versus $\boldsymbol{\lambda_R}$ along the $\boldsymbol{\mu} = -\mathbf{2}$ line, indicating the opening up of the gap by increasing $\boldsymbol{\lambda_R}$. **e-f** BdG spectrum with $\boldsymbol{\mu} = -\mathbf{2}$, (**e**) $\boldsymbol{\lambda_R} = \mathbf{0.1}$ and (**f**) $\boldsymbol{\lambda_R} = \mathbf{0.5}$. (arb. units is an arbitrary unit).

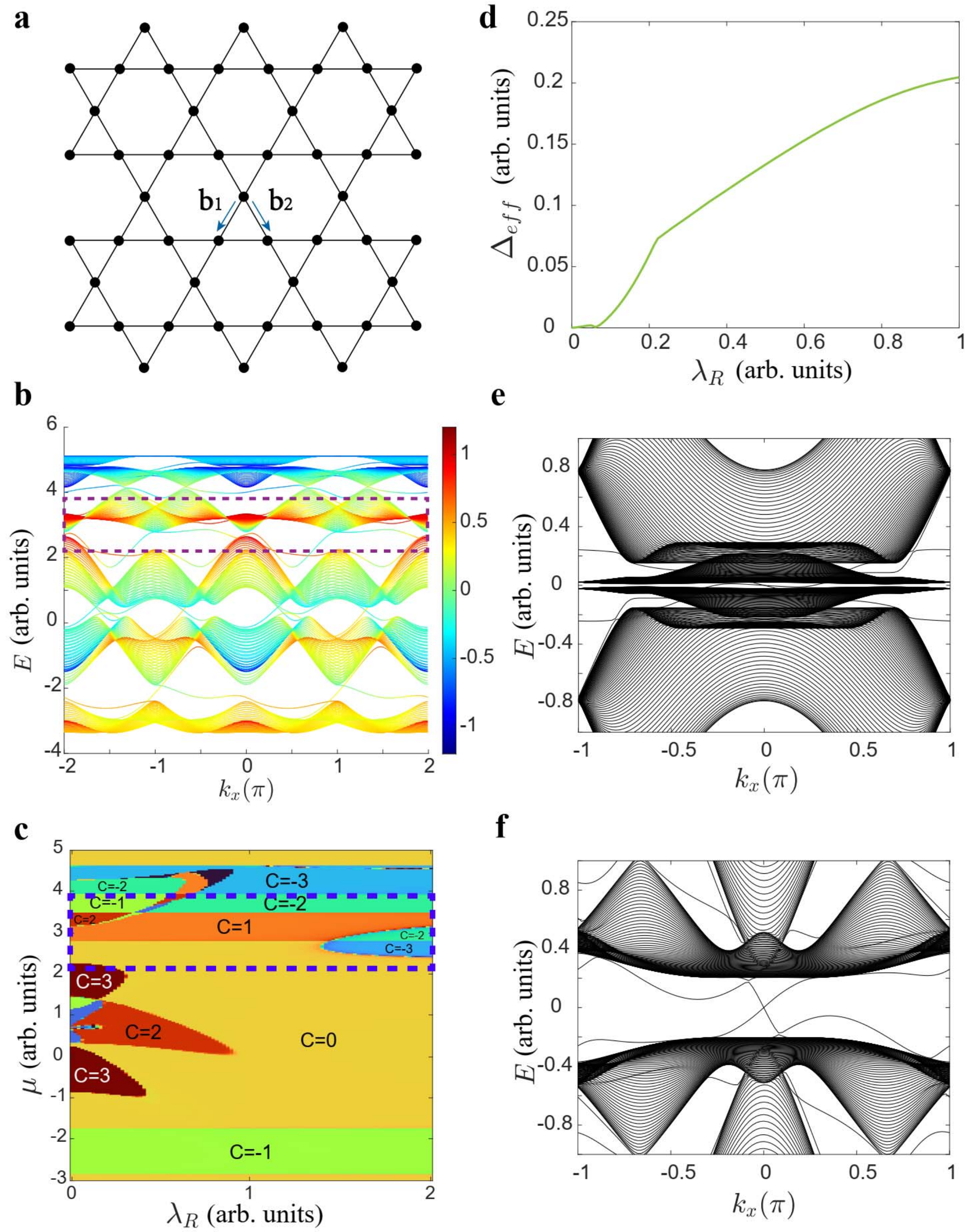

Supplementary Fig. 17 | Theoretical analyses of the emergent superconductivity of the Chern topological magnet/metal heterostructure, considering that the superconductivity takes place in the kagome layer with the weak exchange coupling. a Kagome lattice with unit vectors $\mathbf{b}_1$ and $\mathbf{b}_2$ connecting the nearest bonds in one unit cell. **b** Band structure on the (001) surface without superconductivity, $\boldsymbol{J} = \mathbf{0.75}$, $\boldsymbol{\lambda_{K-M}} = \mathbf{0.1}$, and $\boldsymbol{\lambda_R} = \mathbf{1}$. The colormap represents the spin polarization along the *z* direction. **c** Phase diagram of the Bogoliubov-de Gennes (BdG) Chern number. In the charge-doped region, topological superconductivity emerges near $\boldsymbol{\mu} = \mathbf{3}$. **d** Superconducting gap Δ_{eff} versus $\boldsymbol{\lambda_R}$ with $\boldsymbol{\mu} = \mathbf{3.2}$, indicating the opening up of the gap by increasing $\boldsymbol{\lambda_R}$. **e**-**f** BdG spectrum with $\boldsymbol{\mu} = \mathbf{3.2}$, (**e**) $\boldsymbol{\lambda_R} = \mathbf{0.1}$ and (**f**) $\boldsymbol{\lambda_R} = \mathbf{1}$. (arb. units is an arbitrary unit).

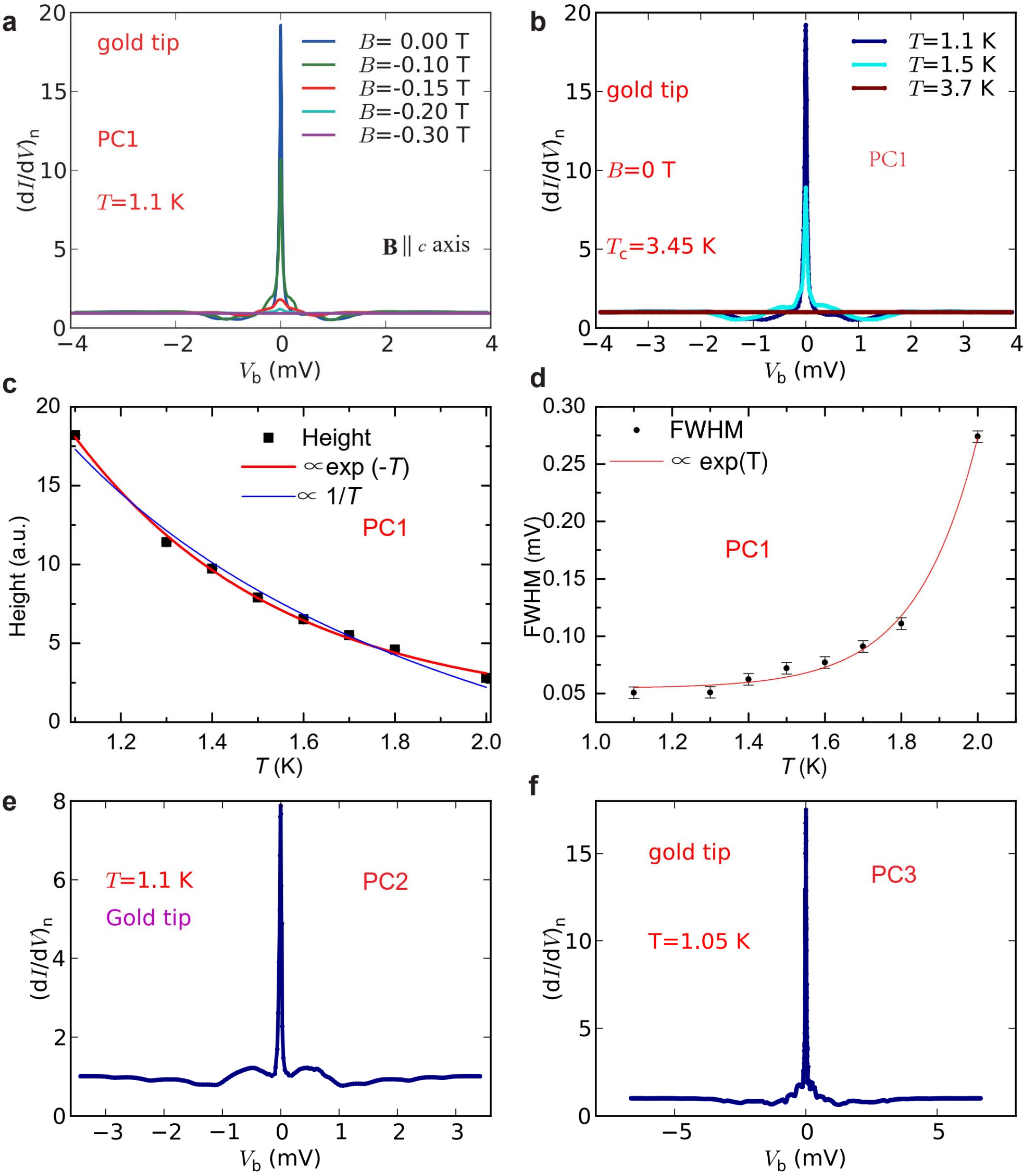

Supplementary Fig. 18 | Zero-bias conductance peaks (ZBCPs) in the PCS of three PC states (marked as PC1, PC2, and PC3) obtained by pressing the Au tip onto the (001) $TbMn_6Sn_6$ surface. a-b The magnetic field and temperature evolution of the ZBCP in the PCS of PC1 state. The ZBCP is suppressed by applying an external magnetic field along the *c*-axis (**a**) or increasing temperature (**b**). **c-d** The extracted height and full width at half maximum (FWHM) of the ZBCP for the PC1 state as a function of temperature. **e-f** Similar ZBCP features observed in the PCS of PC2 (**e**) and PC3 (**f**) states. The resistances for PC1, PC2, and PC3 in the normal state are 4.9 Ω, 2.2 Ω, and 4.2 Ω, respectively. The possibility that the ZBCP is triggered by Joule heating in the contact region can be excluded, and the details can be referred to Supplementary Fig. 19.

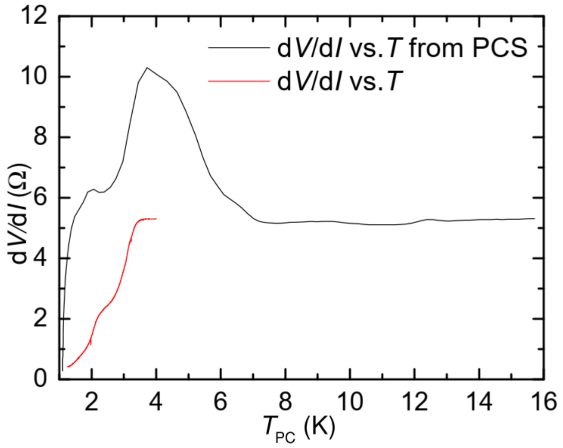

Supplementary Fig. 19 | The black curve shows the resistance versus temperature converted from the zero-field point-contact spectrum in Supplementary Fig. 18a and the red curve shows the zero-bias differential resistance versus temperature (red) measured on the same point contact. The voltage (*V*) in the PCS at 1.1 K in Supplementary Fig. 18a is converted to the temperature (T_{PC}) according to the formula[8]: $T_{PC}^2 = T_{bath}^2 + V^2/4L$, where L = 2.45 × 10^{-8} V^2/K^2 is the Lorenz number, T_{bath} = 1.1 K. The notable difference between the two curves indicates the spectra and zero bias conductance peak shown in Supplementary Fig. 18a are not a result of Joule heating.

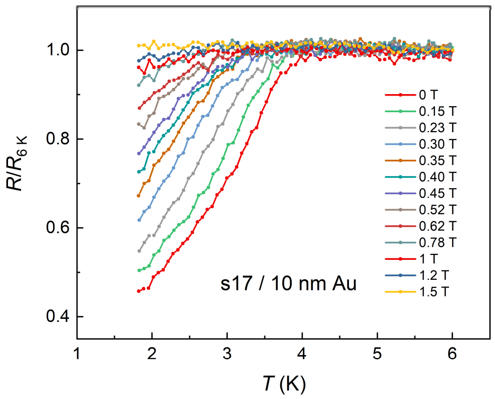

Supplementary Fig. 20 | Temperature dependence of the normalized longitudinal resistance of the $TbMn_6Sn_6$/Au heterostructure s17. The surface of s17 is only ultrasonic cleaned by ethanol and acetone to avoid the possible influence of mechanical force.

Supplementary References

1. Yin, J. et al. Quantum-limit Chern topological magnetism in $TbMn_6Sn_6$. *Nature* **583**, 533-536 (2020).
2. Rashba, E. I. Symmetry of energy bands in crystals of wurtzite type.1 symmetry of bands disregarding spin-orbit interaction. *Sov. Phys.-Solid State* **1**, 368-380 (1959)
3. Nagano, M., Kodama, A., Shishidou, T. & Oguchi, T. A first-principles study on the Rashba effect in surface systems. *J. Phys.: Condens. Matter* **21**, 064239 (2009).
4. LaShell, S., McDougall, B. A. & Jensen, E. Spin splitting of an Au (111) surface state band observed with angle resolved photoelectron spectroscopy. *Phys. Rev. Lett.* **77**, 3419 (1996).
5. Grytsyuk, S. et al. k-asymmetric spin splitting at the interface between transition metal ferromagnets and heavy metals. *Phys. Rev. B* **93**, 174421 (2016).
6. Thouless, D. J., Kohmoto, M., Nightingale, M. P. & Nijs, M. D. Quantized Hall Conductance in a Two-Dimensional Periodic Potential. *Phys. Rev. Lett.* **49,** 405-408 (1982).
7. Wang, J., Zhou, Q., Lian, B. & Zhang, S.-C. Chiral topological superconductor and half-integer conductance plateau from quantum anomalous Hall plateau transition. *Phys. Rev. B* **92**, 064520 (2015).
8. Naidyuk, Y. G. & Yanson, I. K. Point-contact spectroscopy of heavy-fermion systems. *J. Phys.: Condens. Matter.* **10**, 8905 (1998).
9. Lee, W.-C. & Greene, L. H. Recent progress of probing correlated electron states by point contact spectroscopy. *Rep. Prog. Phys.* **79**, 094502 (2016).
10. Andreev, A. F. Thermal conductivity of the intermediate state of superconductors. *Sov. Phys. JETP* **19**,1228 (1964).
11. Deutsche, G. Andreev–Saint-James reflections: a probe of cuprate superconductors. *Rev. Mod. Phys.* **77**, 109 (2005).
12. Daghero, D. & Gonnelli, R. S. Probing multiband superconductivity by point-contact spectroscopy. *Supercond. Sci. Technol.* **23**, 043001 (2010).
13. Wang, H., Ma, L. & Wang, J. Tip-induced or enhanced superconductivity: a way to detect topological superconductivity. *Sci. Bull.* 63, 1141-1158 (2018).
14. Jansen, A. G. M., van Gelder, A. P. and Wyder, P. Point-contact spectroscopy in metals. *J. Phys. C: Solid State Phys.* **13**, 6073 (1980).
15. Blonder, G. E., Tinkham, M. & Klapwijk, T. M. Transition from metallic to tunneling regimes in superconducting microconstructions: Excess current, charge imbalance, and supercurrent conversion. *Phys. Rev. B* **25**, 4515 (1982).
16. Sasaki, S. et al. Kondo effect in an integer-spin quantum dot. *Nature* **405**, 764–767 (2000).
17. Mourik, V. et al. Signatures of Majorana Fermions in Hybrid Superconductor-Semiconductor Nanowire Devices. *Science* **336**, 1003-1007 (2012).
18. Das, A. et al. Zero-bias peaks and splitting in an Al–InAs nanowire topological superconductor as a signature of Majorana fermions. *Nat. Phys.* **8**, 887–895 (2012).
19. Wees, B. J. van, Vries, P. de, Magne´e, P. & Klapwijk, T. M. Excess conductance of superconductor-semiconductor interfaces due to phase conjugation between electrons and holes. *Phys. Rev. Lett.* **69**, 510-513 (1992).

20. Gray, M. J. et al. Evidence for helical hinge zero modes in an Fe-based superconductor. *Nano. Lett.* **19**, 4890-4896 (2019).

21. Chen, C. et al. Atomic line defects and zero-energy end states in monolayer Fe(Te, Se) high-temperature superconductors. *Nat. Phys.* **16,** 536–540 (2020).
